# TET-Catalyzed 5-Hydroxymethylation Precedes *HNF4A* Promoter Choice during Differentiation of Bipotent Liver Progenitors

**DOI:** 10.1016/j.stemcr.2017.05.023

**Published:** 2017-06-22

**Authors:** Pierre-Benoit Ancey, Szilvia Ecsedi, Marie-Pierre Lambert, Fazlur Rahman Talukdar, Marie-Pierre Cros, Denise Glaise, Diana Maria Narvaez, Veronique Chauvet, Zdenko Herceg, Anne Corlu, Hector Hernandez-Vargas

**Affiliations:** 1 Epigenetics Group, International Agency for Research on Cancer (IARC), 150 Cours Albert Thomas, 69008 Lyon, France; 2 MTA-DE Public Health Research Group, University of Debrecen, 4028 Debrecen, Hungary; 3 Epissage alternatif et progression tumorale, Centre de Recherche en Cancérologie de Lyon (CRCL), 28 rue Laennec, 69008 Lyon, France; 4 Inserm, Inra, UBL, Nutrition Metabolism and Cancer (NuMeCan), 35033 Rennes Cedex 9, France; 5 Human Genetics Laboratory, Department of Biological Sciences, Universidad de Los Andes, Cr. 1 No. 18A-10 Building M1-2 Floor, Bogotá 110321, Colombia

**Keywords:** liver progenitor, DNA methylation, 5mC, 5hmC, HNF4A

## Abstract

Understanding the processes that govern liver progenitor cell differentiation has important implications for the design of strategies targeting chronic liver diseases, whereby regeneration of liver tissue is critical. Although DNA methylation (5mC) and hydroxymethylation (5hmC) are highly dynamic during early embryonic development, less is known about their roles at later stages of differentiation. Using an in vitro model of hepatocyte differentiation, we show here that 5hmC precedes the expression of promoter 1 (P1)-dependent isoforms of *HNF4A*, a master transcription factor of hepatocyte identity. 5hmC and HNF4A expression from P1 are dependent on ten-eleven translocation (TET) dioxygenases. In turn, the liver pioneer factor FOXA2 is necessary for TET1 binding to the P1 locus. Both FOXA2 and TETs are required for the 5hmC-related switch in HNF4A expression. The epigenetic event identified here may be a key step for the establishment of the hepatocyte program by HNF4A.

## Highlights

• 5hmC marks *HNF4A* P1 promoter previous to terminal hepatocyte differentiation
• TET1-dependent 5hmC is required to activate P1-driven HNF4A expression
• Pioneer factor FOXA2 is necessary for TET1 binding to P1 locus
• HNF4A promoter switch is reversible upon hepatocyte dedifferentiation

## Graphical Abstract

**Figure:**
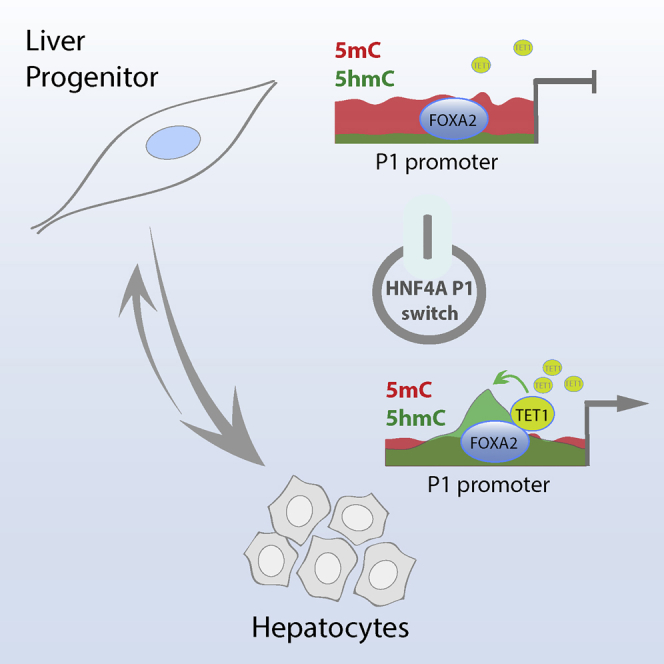

## Teaser

Hepatocyte identity is impaired in various human chronic liver diseases. Methylation and hydroxymethylation dynamically mark the DNA during development. As shown by Hernandez-Vargas and colleagues, discrete changes in these marks also play a role in establishing hepatocyte identity. Specifically, hydroxymethylation of the hepatocyte promoter *HNF4A* signals a key switch in expression. Because of its reversibility, this switch is susceptible to experimental manipulation.

## Introduction

Contrary to other human organs, the cellular hierarchy of the liver is still a matter of debate. Hepatic progenitor cells (HPCs) are able to supply two types of liver epithelial cells, hepatocytes and cholangiocytes, during cellular turnover. Mature hepatocytes retain the capacity to regenerate the liver cell pool after tissue loss (e.g., partial hepatectomy) (Miyajima et al., 2014). However, when liver tissue is damaged due to chronic pathology (e.g., cirrhosis and/or hepatocellular carcinoma) progenitor-like cells accumulate, in a process known as ductular reaction. Recent evidence suggests that these HPCs are created by hepatocyte dedifferentiation (Mu et al., 2015), although their fate and regenerative capacity remain the subject of controversy. Understanding the mechanisms that underlie this (de)differentiation has important implications for regenerative medicine (Khan et al., 2010).

DNA methylation (i.e., 5-methylcytosine [5mC]) is known to play an important role in early development, whereby waves of demethylation and remethylation take place as part of a genome-wide shaping of the chromatin. The involvement of 5mC in stem cell differentiation has been supported by several reports of enhanced differentiation after treatment with demethylating agents (Mohn et al., 2008). Additional evidence comes from reprogramming experiments (Kim et al., 2010, Lister et al., 2011, Ruiz et al., 2012), including attempts to directly convert hepatic cell lineages by expression of defined transcription factors (TFs) (Vallier, 2014, Yu et al., 2013, Zhu et al., 2014). Known difficulties in achieving hepatic stem/progenitor cell maintenance may be due to the presence of other important mechanisms in the differentiation process. In this sense, it has been shown that oxidized forms of 5mC, and especially DNA hydroxymethylation (i.e., 5-hydroxymethylcytosine [5hmC]), have a role in development and organogenesis (Sun et al., 2014a). However, less is known about the role of 5mC and 5hmC in processes of differentiation taking place in adult tissues, such as those related to tissue renewal from progenitor cells.

To identify key events involved in liver progenitor cell differentiation, we profiled 5mC marks at the genome-wide level and found discrete loci progressively changing methylation during this process. We focused on the most significant of these, demethylation of the *HNF4A* locus, a master TF of hepatocyte identity. This locus is marked by 5hmC in bipotent progenitors poised for differentiation, and enables a switch in HNF4A isoform expression. This redistribution of 5hmC marks within *HNF4A* is observed in several in vitro systems and during the transition from fetal to adult human liver.

## Results

### HNF4A Is Progressively Demethylated during Hepatocyte Differentiation of HepaRG Progenitors

**Figure 1:**
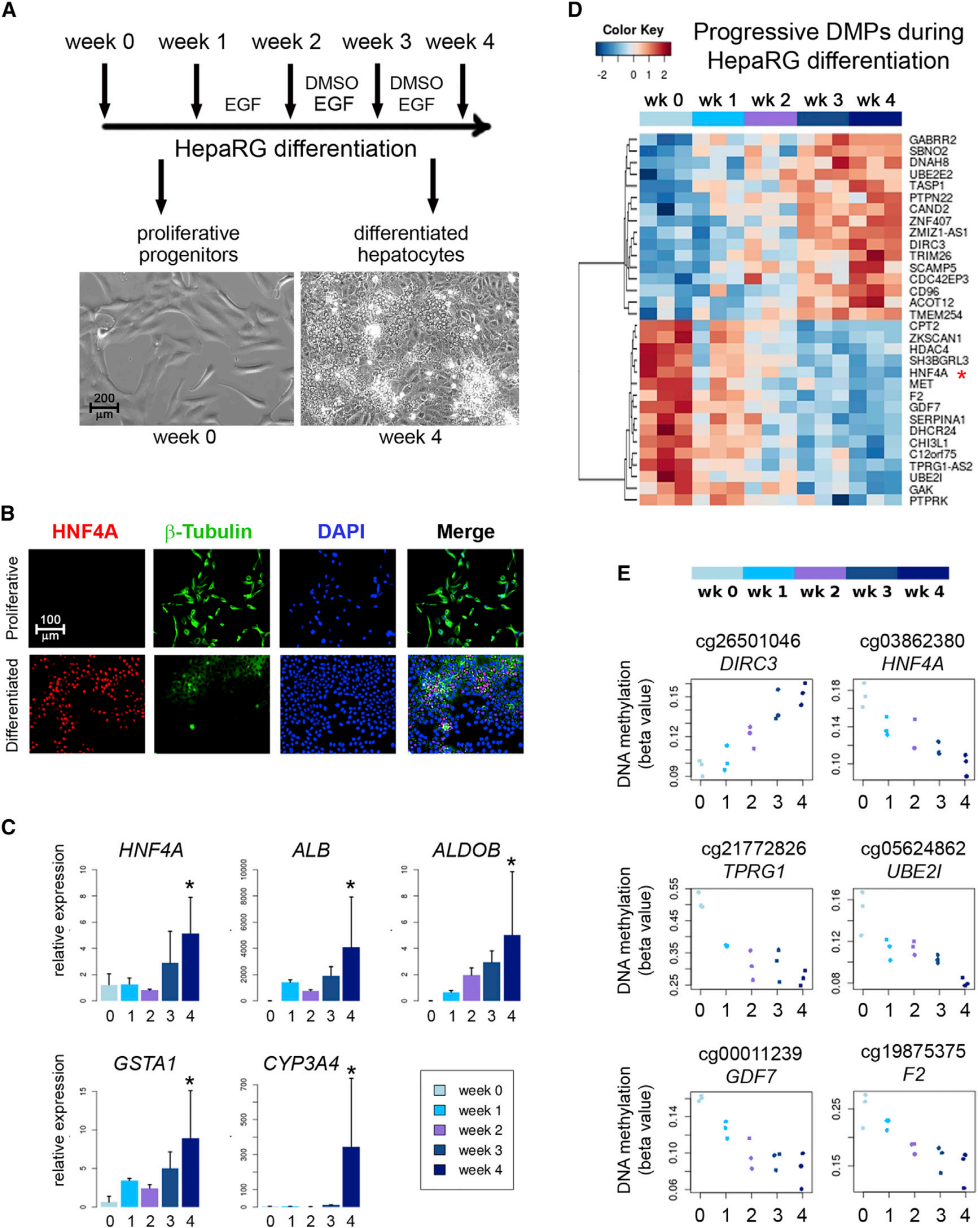
Genome-wide 5mC Profiling of HepaRG Progenitors during Differentiation (A) Protocol of HepaRG differentiation. Samples were taken at each of the indicated time points for genome-wide methylation analyses. Example phase contrasts are shown for proliferative HepaRG (left panel) and differentiated HepaRG (right panel). (B) Immunofluorescence comparing progenitors (upper panels) and differentiated (lower panels) HepaRG cells. Stainings are shown for β-tubulin and HNF4A (all isoforms), with DAPI for nuclear staining. (C) Expression of markers of differentiated hepatocytes by qRT-PCR at different time points. All genes were significantly upregulated at the hepatocyte stage (^∗^p < 0.05). Results are presented as means ± SD. (D) Heatmap of differentially methylated positions (DMPs) displaying progressive changes during HepaRG differentiation, with a minimum change of 5% methylation between week 0 and week 4 (Table 1). (E) Example stripcharts of the top most significant DMPs shown in (D). All genome-wide results and validations (shown in Figure S1) were performed in at least three independent biological replicates.

We used a well-established 4-week protocol for in vitro differentiation of the bipotent human progenitor cell line HepaRG toward hepatocyte-like cells (Figure 1A) (Cerec et al., 2007, Gripon et al., 2002). Efficiency of hepatocyte differentiation was validated by studying the typical changes in morphology and gene expression involved in this process. The former includes the emergence of small polygonal cells with increased refraction and granularity, organized in well-delineated trabeculae separated by bright canaliculi-like structures (Figure 1A). The latter includes the increased nuclear staining of HNF4A (Figure 1B), and progressive expression of markers of hepatocyte metabolic activity (i.e., *HNF4A*, albumin, aldolase B, glutathione S-transferase α, and *Cyp3A4*) by qRT-PCR (Figure 1C).

**Figure 2:**
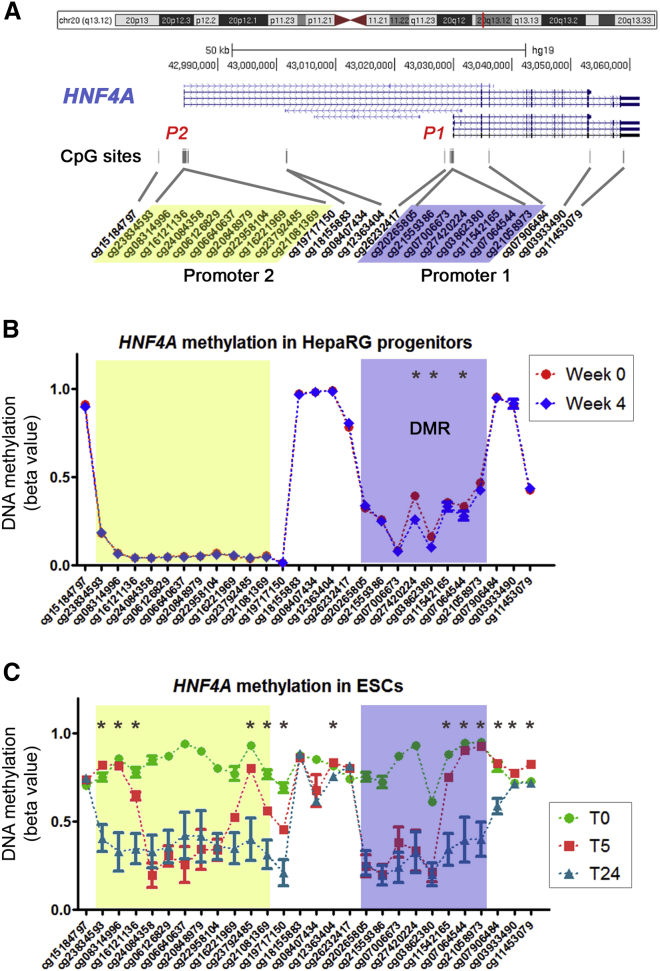
Region-Level 5mC in HepaRG Progenitors and ESCs (A) UCSC screenshot of the *HNF4A* locus. Alternative promoters (P1 and P2) and all informative CpG sites within *HNF4A* are shown in their approximate location. (B) Infinium 450k methylation data (HepaRG progenitors [in red] versus their differentiated progeny [in blue]) was plotted for all CpG sites shown in (A). The identified differentially methylated region (DMR) corresponding to the *HNF4A* promoter 1 (P1) is highlighted with a purple-blue rectangle while the P2 promoter is shown in yellow. (C) Infinium 450k data during differentiation of embryonic stem cells (ESCs) toward hepatocytes. Cells were obtained at different time points (days 0 [green], 5 [red], and 24 [blue]), as previously described (Wilson et al., 2015). 5mC values for all HNF4A CpG sites, as shown in (A), highlighting *HNF4A* promoters P1 (blue) and P2 (yellow). Similar analysis for iPSCs is shown in Figure S2. ^∗^p value < 0.05, t test.

Samples selected for methylome-wide analysis had between 60% and 80% hepatocytes at week 4 of differentiation, as assessed by HNF4A staining (Figure 1B). DNA was extracted every week and processed for bead array methylation following standard protocols. After data preprocessing and quality control, we performed differential methylation analysis at the site and region levels, modeling time as a continuous variable. In both analyses, the most significant change in methylation (lowest p values) mapped to the promoter 1 (P1) of *HNF4A* (Tables 1 and S1). In addition, changes of at least 5% methylation were observed in 32 sites, displaying progressive increase (n = 16) or decrease (n = 16) of methylation throughout the differentiation process (Figure 1D). Sites progressively hypomethylated were significantly enriched in HNF4A targets (10 out of 16) (Figure 1E and Table 1). *HNF4A* P1 demethylation reached up to 13% change on one of the eight CpG sites (cg27420224 in Figures 2A and 2B) spanned by the differentially methylated region (DMR), a finding validated using quantitative bisulfite pyrosequencing (Figure S1A). Of note, there was no differential methylation in other genes known to increase expression during differentiation (such as those shown in Figure 1C) (data not shown). Demethylation of two additional loci (*F2* and *GAK*, out of four assays tested) was also validated by pyrosequencing (Figure S1B).

**Table 1:** Differentially Methylated Positions

Target_ID	Symbol	p Value	Log2 Fold Change	Distance	Nearest Transcript
cg03862380	*HNF4A*	1.71 × 10^−6^	−0.22	0	uc002xly.4
cg00011239	*GDF7*	4.52 × 10^−6^	−0.28	4,969	uc002rdz.1
cg21772826	*TPRG1*	9.51 × 10^−6^	−0.34	0	uc031scu.1
cg21646082	*CEP85*	1.16 × 10^−5^	−0.30	0	uc001blu.3
cg15894315	*DHCR24*	1.8 × 10^−5^	−0.23	0	uc010ooi.1
cg26501046	*DIRC3*	1.87 × 10^−5^	0.19	0	uc002vgn.2
cg05624862	*UBE2I*	2.21 × 10^−5^	−0.21	9,495	uc002clc.2
cg19875375	*F2*	2.31 × 10^−5^	−0.26	75	uc001ndf.4
cg02214698	*GAK*	4.23 × 10^−5^	−0.30	0	uc003gbl.4
cg10547843	*AZGP1P1*	4.34 × 10^−5^	−0.27	10,103	uc003usj.3
cg24621042	*SERPINA1*	4.89 × 10^−5^	−0.20	245	uc001ycy.4
cg24632480	*TMEM254*	6.27 × 10^−5^	0.38	11,774	uc001kbn.5
cg02062466	*C12orf75*	7.71 × 10^−5^	−0.20	12,105	uc001tlh.4
cg07423149	*CHI3L1*	9.1 × 10^−5^	−0.15	323	uc001gzi.2
cg00351152	*CAND2*	9.4 × 10^−5^	0.27	1,152	uc003bxj.2
cg26517584	*SBNO2*	0.000106	0.28	0	uc002lrk.4
cg01701819	*SCAMP5*	0.000111	0.23	50	uc002azk.2
cg09664216	*HDAC4*	0.000115	−0.23	0	uc010fyz.1
cg15933457	*CDC42EP3*	0.000117	0.21	104,010	uc031roa.1
cg02794920	*UBE2E2*	0.00013	0.33	1,351	uc010hfc.2
cg02619107	*ZNF407*	0.000136	0.27	0	uc010xfc.2
cg11319403	*MET*	0.000145	−0.22	0	uc011knj.2
cg04581728	*DNAH8*	0.00016	0.20	0	uc003ooe.2
cg03525433	*CD96*	0.000166	0.27	1,107	uc003dxv.3
cg03601886	*CPT2*	0.000182	−0.27	12,881	uc001cvb.4
cg11972176	*TASP1*	0.000192	0.22	0	uc010zri.1
cg07130494	*PTPRK*	0.000198	−0.32	0	uc003qbj.3
cg16580289	*GABRR2*	0.000208	0.31	288	uc003pnb.3
cg00041401	*AP4B1-AS1*	0.000215	0.29	0	uc001eds.3
cg05006030	*RNU5E-1*	0.000221	0.19	0	uc003khl.4
cg06891775	*ZMIZ1-AS1*	0.000243	0.19	0	uc001jzx.3
cg19397765	*TRIM26*	0.000246	0.16	492	uc003nps.3

Known HNF4a targets (based on ENCODE Transcription Factor ChIP-seq data 2015) are underlined. Distance: distance (base pairs) to the closest transcription start site.

See also Table S1 for differentially methylated regions (DMRs).

### HNF4A Is Progressively Demethylated during Hepatocyte Differentiation from ESCs and iPSCs

The DMR identified in *HNF4A* included eight CpG sites and extended for 529 bp along the P1 promoter (Figures 2A and 2B; Table S1), which controls the expression of at least four different *HNF4A* isoforms (Figure 2A). In contrast, there was no significant change in methylation in the upstream (P2) *HNF4A* promoter that controls the expression of so-called P2-driven *HNF4A* isoforms (Figure 2B). Although significant at the region level, only three CpG sites within the DMR display a difference higher than 5% between weeks 0 and 4 of differentiation (shown with an asterisk in Figure 2B). To explore potential differences at earlier stages of differentiation and to validate our results in two independent in vitro systems, we took advantage of published genome-wide 5mC data (Wilson et al., 2015). The first model involves differentiation from human embryonic stem cells (ESCs) while the second model is based on differentiation from human induced pluripotent stem cells (iPSCs). In both cases, 5mC was studied at the stem cell stage (T0), after establishment of definitive endoderm progenitors (T5) and at the end of the hepatocyte differentiation process (T24). Of note, unsupervised clustering based on all CpG sites mapped to *HNF4A* was able to discriminate all samples based on their developmental stage (i.e., T0, T5, or T24), and regardless of the in vitro model system (Figure S2A).

When visualizing the HNF4A locus in more detail during ESC differentiation, we observed a global demethylation at the first differentiation transition, from stem to definitive endoderm cells (T0 versus T5 in Figure 2C). However, several regions seemed to be preserved from this global demethylation, including part of the P1 promoter (as described above) and two discrete loci upstream and downstream of the P2 promoter (Figure 2C). Also in the ESC model, 13 CpG sites (three of them within the HNF4A P1 DMR) were differentially demethylated (p < 0.05, at least 5% change in methylation) during the final T5-T24 transition (Figure 2C), in line with our results in HepaRG progenitors. Similar findings were obtained with the analysis of 5mC data from iPSCs (Figure S2B). Of note, the last stage of differentiation (T5 to T24) matches the terminal differentiation from bipotent progenitors, and is comparable with the HepaRG system. In this sense, complete demethylation of the master hepatocyte TF *HNF4A* locus is a late event in three different in vitro systems of hepatocyte differentiation (i.e., HepaRG, ESCs, and iPSCs).

### HNF4A P1 Demethylation Is Associated with a Reversible Isoform Expression Switch

**Figure 3:**
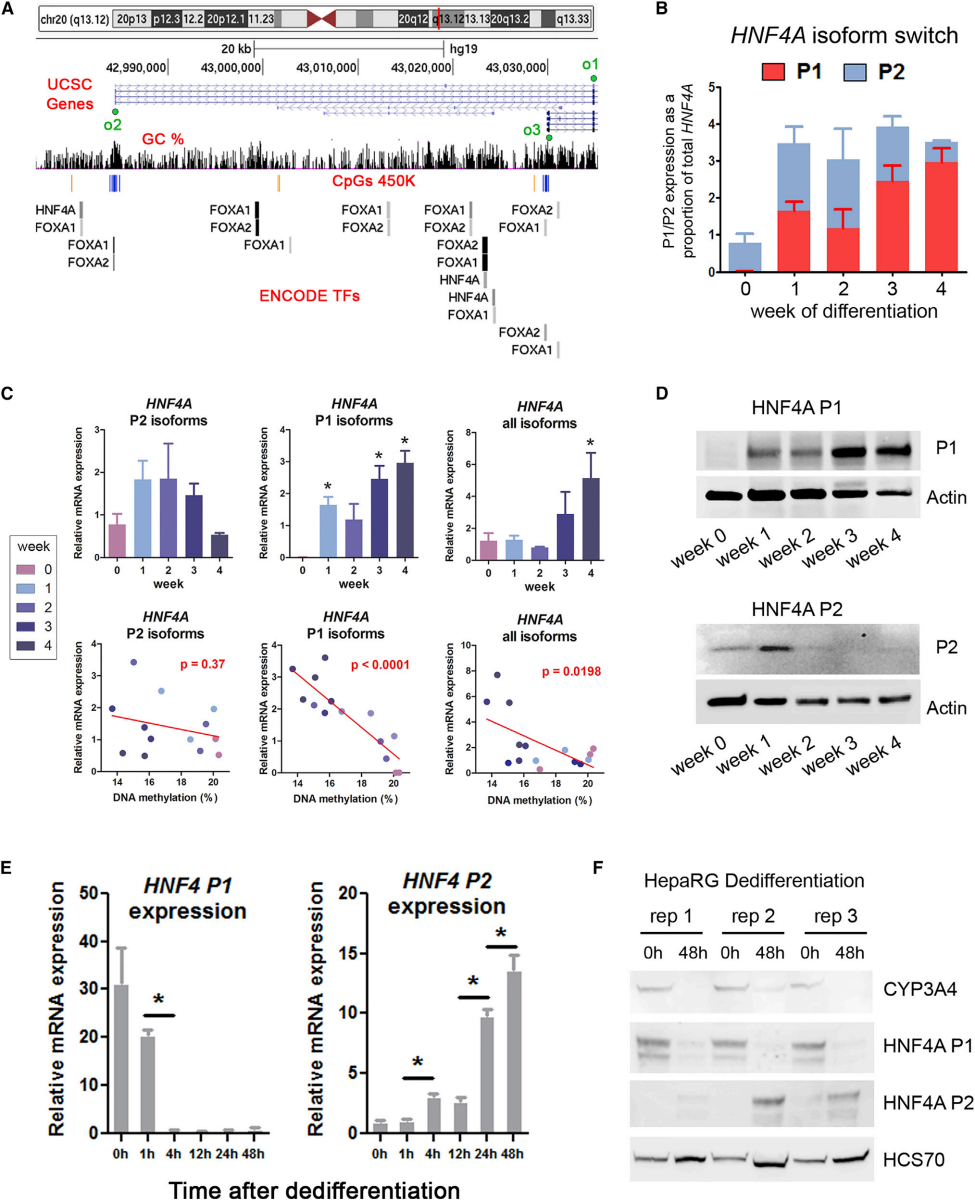
Reversible HNF4A Isoform Switch during Hepatocyte Differentiation (A) UCSC screenshot of the *HNF4A* locus. Oligos designed for qRT-PCR (o1, o2, and o3) are shown in their approximate location, with o3 matching the differentially methylated region (DMR) at the P1 promoter. ENCODE ChIP-seq peaks are shown for FOXA1/2 and HNF4A (UCSC tracks for HepG2 cells). (B) Expression of mRNAs from P1 and P2 isoforms is shown as a proportion of the total *HNF4A* expression at each time point of differentiation. (C) mRNA expression and expression/methylation correlations for the primers indicated in (A) are shown in upper and lower panels, respectively. Expression values are normalized to the first time point (week 0) for each qRT-PCR. p Values for each correlation (Pearson) are included on the lower panels. (D) Western blots with HNF4A isoform-specific antibodies at different time points of HepaRG differentiation. Actin was used as housekeeping protein. (E and F) Following differentiation, hepatocytes were selected and plated at low confluence to induce dedifferentiation, and P1- and P2-isoform expression was assessed at different time points by qRT-PCR (E) and western blot (F). HCS70 was used as housekeeping protein, and quantifications can be found in Figure S3. Results in (E) are presented as means ± SD. All expression data were obtained from three independent biological replicates (labeled as “rep” in F). ^∗^p < 0.05, t test.

Because of the key role of HNF4A in hepatocyte differentiation (Parviz et al., 2003, Watt et al., 2003), we studied the potential consequence of demethylation at the expression level. qRT-PCR assays specific for the products of P1 and P2 (Figure 3A) showed that P1-dependent isoforms accounted for the increased *HNF4A* expression (Figure 3B), consistent with previous observations (Cerec et al., 2007). Moreover, expression from P1 was highly negatively correlated with DMR demethylation compared with the expression from the P2 promoter (Figure 3C). The switch in mRNA expression from P2- to P1-driven isoforms during the progenitor-hepatocyte transition was also seen at the protein level, by using antibodies specific for P1- and P2-driven isoforms of HNF4A (Figures 3D and S3). While P2-driven protein decreases abruptly after the first week of progenitor differentiation, P1-driven HNF4A (which is not detectable at the progenitor stage) becomes progressively accumulated throughout the differentiation process. Although in general mRNA- and protein-based assays correlate well, we cannot rule out that post-translational modifications also take place. Indeed, it was shown that translational regulation may play an important role in the same HepaRG model of differentiation (Parent and Beretta, 2008).

Next, we studied the stability of this process by inducing dedifferentiation from the hepatocyte stage using a well-established protocol for dedifferentiation based on the selection of hepatocytes at day 30 of differentiation and replating at lower confluence (Dubois-Pot-Schneider et al., 2014). Under these conditions, transcriptional programs are reverted to the progenitor stage as soon as 24 hr after replating. In this way we observed a progressive shutdown of *HNF4A* expression from the P1 promoter and re-expression of P2-driven isoforms (Figure 3E), opposite to the pattern observed during progenitor differentiation.

These data uncovered a P2-to-P1 HNF4A expression switch in HepaRG progenitors, detectable as early as 1 week after inducing differentiation toward hepatocytes and reversible as early as 1 day after dedifferentiation.

### HNF4A P1 Hydroxymethylation Precedes Terminal Hepatocyte Differentiation

**Figure 4:**
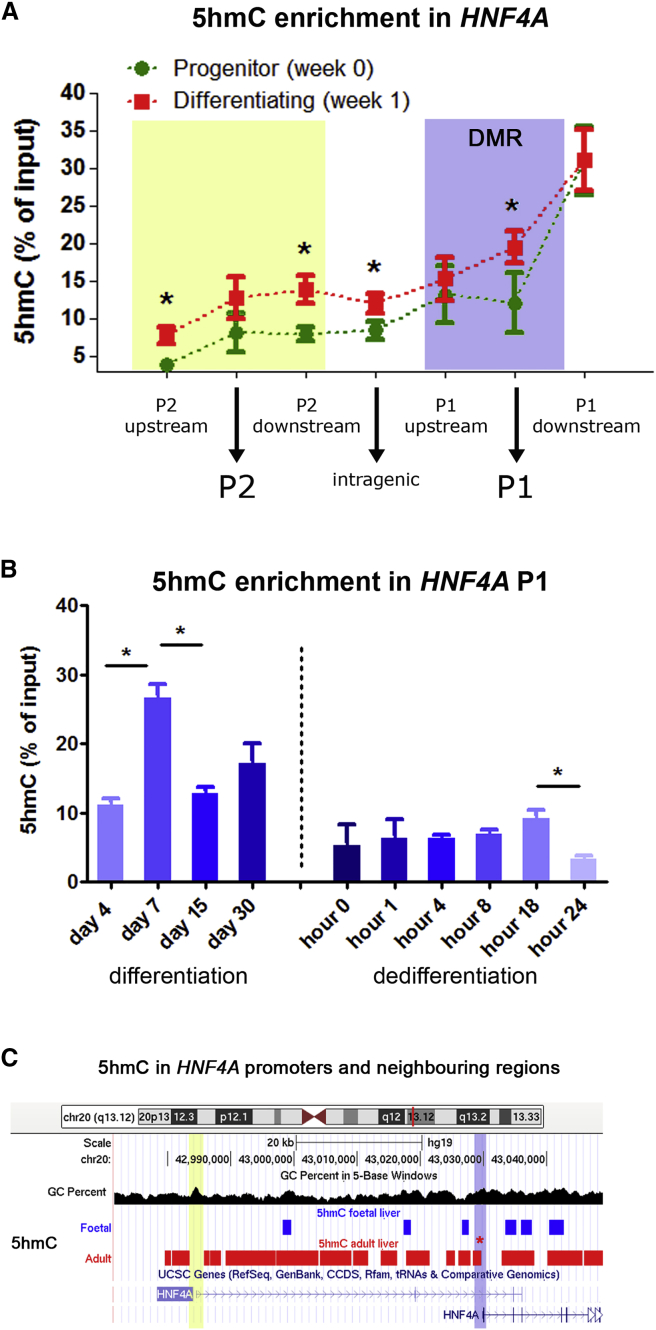
Hydroxymethylation of *HNF4A* P1 Promoter (A) 5-Hydroxymethylcytosine level (5hmC) was assessed after immunoprecipitation with anti-5hmC antibody (hMedIP). hMedIP was used to map 5hmC along the *HNF4A* gene, including its two promoter regions P1 (blue) and P2 (yellow). DMR, differentially methylated region. Results (percent of input) are shown at the progenitor level and after 1 week of differentiation. (B) Kinetics of 5hmC was assessed by hMedIP for *HNF4A* P1 promoter until full hepatocyte differentiation (left panel) and following dedifferentiation (as shown in Figure 3E). DNA was extracted for hMedIP at different time points of differentiation and dedifferentiation up to 24 hr after replating. (C) 5hmC detection blocks for adult and fetal livers were downloaded directly from the corresponding publication (Ivanov et al., 2013). UCSC tracks were used to visualize 5hmC blocks from fetal (blue) and adult (red) tissues overlapping the *HNF4A* locus. One of the regions enriched in adult liver overlapped with the DhMR identified in HepaRG cells (red asterisk). 5mC data for fetal versus adult livers is shown in Figure S4. Error bars represent mean ± SD from three independent experiments. ^∗^p < 0.05, t test.

Demethylation can be the result of a cell cycle-dependent deficiency in the addition of methyl residues to nascent DNA strands (passive demethylation) or the consequence of progressive oxidation of methyl-cytosines by ten-eleven translocation (TET) dioxygenases (active demethylation) (Schübeler, 2015). In favor of the second possibility, HepaRG progenitors reach confluence after 1 week of culture and are therefore limited in their proliferative status, although additional cell divisions cannot be ruled out. In addition, expression of *TET1* and *TET2* was increased during the first week of differentiation, matching the time of highest overexpression of P1-dependent *HNF4A* isoforms, although only *TET1* overexpression reached statistical significance (Figure S4A). We therefore assessed the presence of 5hmC, which reflects the enzymatic activity of TETs, using 5hmC immunoprecipitation (hMedIP). We mapped 5hmC at different locations within the *HNF4A* locus, including its two promoters (with primers up- and downstream of the original 5mC data), and one intragenic locus between both promoters (Figure 4A). We found a global increase in 5hmC after 1 week of progenitor differentiation (Figure 4A). Increased 5hmC was statistically significant at the P1 promoter and the selected loci upstream and downstream of P2. This matches the profile of late demethylation observed in ESCs (Figure 2C) and iPSCs (Figure S2B), and establishes *HNF4A* as a differentially hydroxymethylated region (DhMR).

Of note, the observed increase in 5hmC was at least one order of magnitude higher than the decrease in 5mC at the HNF4A P1 locus, a finding validated with a base-resolution technique (i.e., oxidative bisulfite [oxBS]) (Figure S4B). This led us to explore in more detail the kinetics and dynamics of 5hmC during differentiation. Using hMedIP, we confirmed an increase in 5hmC at the P1 promoter as early as 1 week of differentiation (Figure 4B). However, we observed a reduced occupancy of 5hmC during the subsequent weeks of differentiation, up to the hepatocyte stage (day 30). This suggests that a 5hmC marks this locus for demethylation in a transient fashion. Using the same protocol for dedifferentiation described above (Figure 3E), we found that 5hmC content is further reduced, reaching its lowest P1 occupancy after 24 hr (Figure 4B).

A switch in HNF4A promoter usage has been described in fetal versus adult liver (Torres-Padilla et al., 2001). To study a potential role of 5mC/5hmC in this process, we extracted published data corresponding to all CpG sites mapping *HNF4A* in human fetal and adult liver (Bonder et al., 2014). 5mC was able to discriminate fetal and adult liver tissues (Figure S4C). Globally there was a significant reduction in 5mC content along the HNF4A locus during the fetal-to-adult transition (Figure S4D). However, the pattern was more complex for the P1 promoter, with upstream hypermethylation and downstream hypomethylation (Figure S4D). In addition, by reanalyzing 5hmC data for the same locus (Ivanov et al., 2013), we found that *HNF4A* is among the genes that gain 5hmC in the fetal-to-adult transition (Figure 4C). Although 5hmC increase seems global along the *HNF4A* locus (matching the global 5mC reduction shown in Figure S4D), one of the enriched regions overlaps with the HepaRG DhMR (Figure 4C).

In summary, 5hmC precedes P1 expression in terminal hepatocyte differentiation. Transition from fetal to adult human liver is also characterized by overall increase in *HNF4A* 5hmC content. Although they do not fully overlap, 5hmC changes observed in vivo and in vitro suggest that the HNF4A P1 DhMR region represents a functionally dynamic locus.

### Pioneer TF FOXA2 Colocalizes with TET1 at the HNF4A P1 Promoter

**Figure 5:**
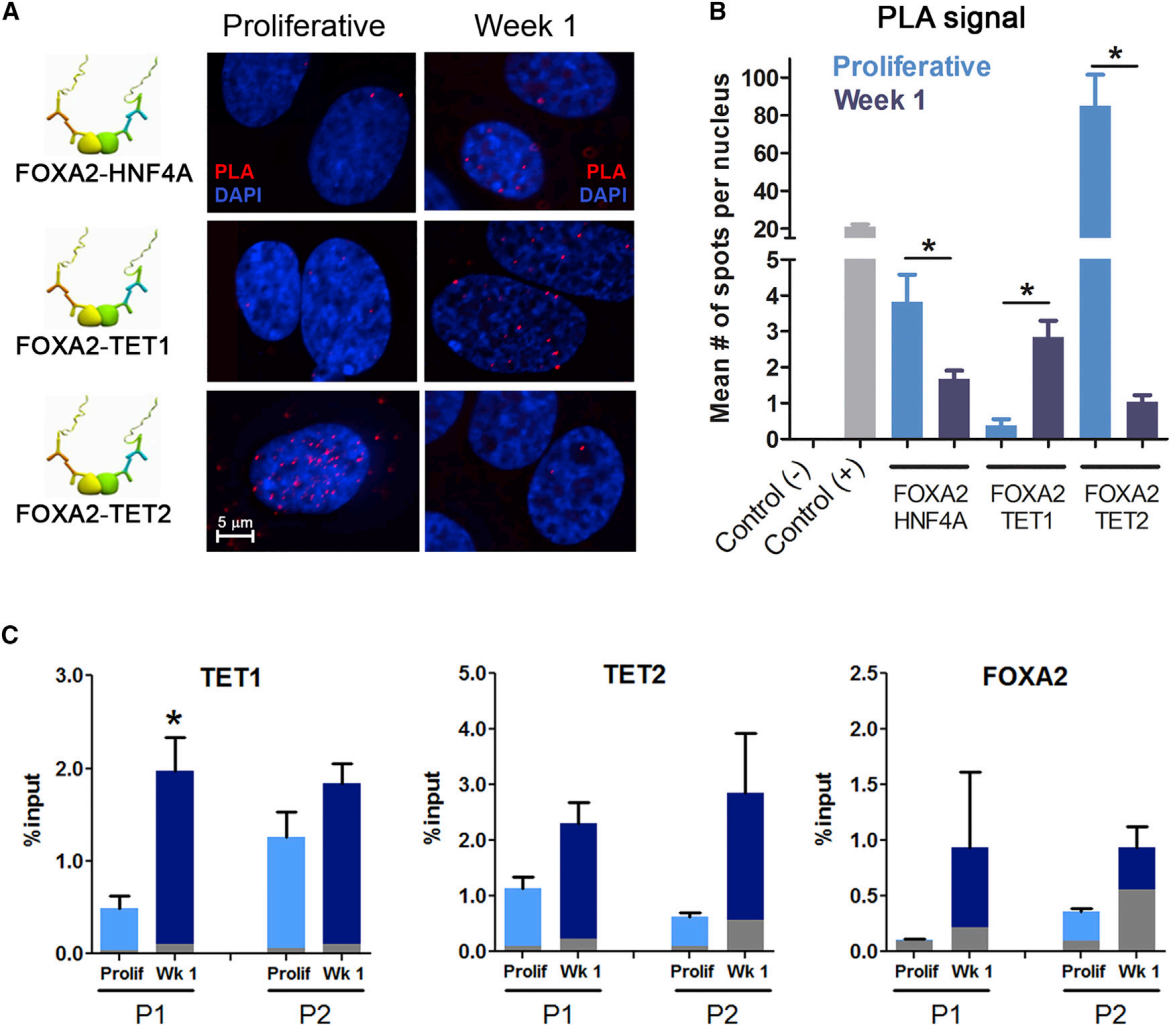
TET1 and FOXA2 Colocalize at the *HNF4A* P1 Locus Proximity ligation assays (PLA) were performed at the progenitor stage (week 0) and after 1 week of differentiation to detect the interaction between FOXA2 and different interactants. (A) PLA assays for the proximity between FOXA2 and HNF4A (known interaction) and FOXA2 and TET proteins (TET1, and TET2). Representative images are shown. (B) Quantification of PLA spots (average number of spots per nucleus). Additional controls include an assay without secondary antibody (negative control) and two antibodies against FOXA2 from different species (positive control). (C) Chromatin immunoprecipitation (ChIP) was performed with antibodies against TET hydroxylases and FOXA2 at the progenitor stage and after 1 week of differentiation. qPCRs were performed for *HNF4A* P1 and P2 promoters, as depicted in Figure 3A. Gray bars represent background IgG signal. PLA and ChIP assays were performed in two and three biological replicates, respectively. Representative results are shown. Results are presented as means ± SD. ^∗^p < 0.05, t test.

The data described above support a model in which a liver bipotent progenitor is poised for 5hmC at the *HNF4A* P1 promoter by TET proteins at an early step of terminal hepatocyte differentiation. This raises the question as to which mechanisms confer genomic specificity to the activity of TETs. By inspecting available chromatin immunoprecipitation (ChIP) data of the *HNF4A* P1 locus, we found that the identified DhMR overlaps with a regulatory region characterized by putative binding for several TFs (Figure 3A). Among these, we found a specific binding site for the hepatocyte pioneer factor FOXA2 (hepatocyte nuclear factor 3β, or HNF3-B) (Iwafuchi-Doi and Zaret, 2014), between 306 and 7 bp upstream of the P1 transcription start site. Because of its known expression at the liver progenitor stage (Cerec et al., 2007), its role in differentiation, and its overlap with the *HNF4A* DhMR, we selected FOXA2 as a reasonable candidate involved in *HNF4A* P1 transcription. We used proximity ligation assays (PLA) to study a potential interaction between FOXA2 and TET proteins, first using HNF4A itself as a positive control, as FOXA2 has been shown to interact with HNF4A during hepatocyte differentiation (Alder et al., 2014, Wallerman et al., 2009). After validating such interaction (Figure 5A, upper panel), we next studied the proximity between FOXA2 and TET proteins. Although no signal was observed at the progenitor stage, we found that FOXA2 colocalizes with TET1 (Figure 5A, middle panel) after 1 week of differentiation, a finding that could be explained by increased *TET1* expression. However, an opposite trend was observed for FOXA2/TET2 colocalization (i.e., a strong signal observed at the progenitor stage is absent after the first week of differentiation) (Figure 5A, bottom panel). As the expression kinetics of both TET proteins is similar (Figure S4A), our findings suggest that FOXA2 sequentially interacts with TET2 and TET1 during the first week of liver progenitor differentiation.

Supporting a role for TET1 in HepaRG differentiation, we observed a significant enrichment of this protein at the *HNF4A* P1 DhMR, using ChIP (Figure 5C). Consistent with PLA results, TET1 binding was significantly increased at the P1 promoter after 1 week of differentiation (Figure 5C), while no changes were observed in TET2 occupancy. FOXA2 occupancy was detected but unchanged at the P1 promoter, consistent with its pioneer role. Of note, no significant changes were observed for TETs or FOXA2 at the P2 promoter, although TET2 occupancy was higher at week 1.

### FOXA2 and TETs Are Required for the 5hmC-Related Switch in HNF4A Expression

**Figure 6:**
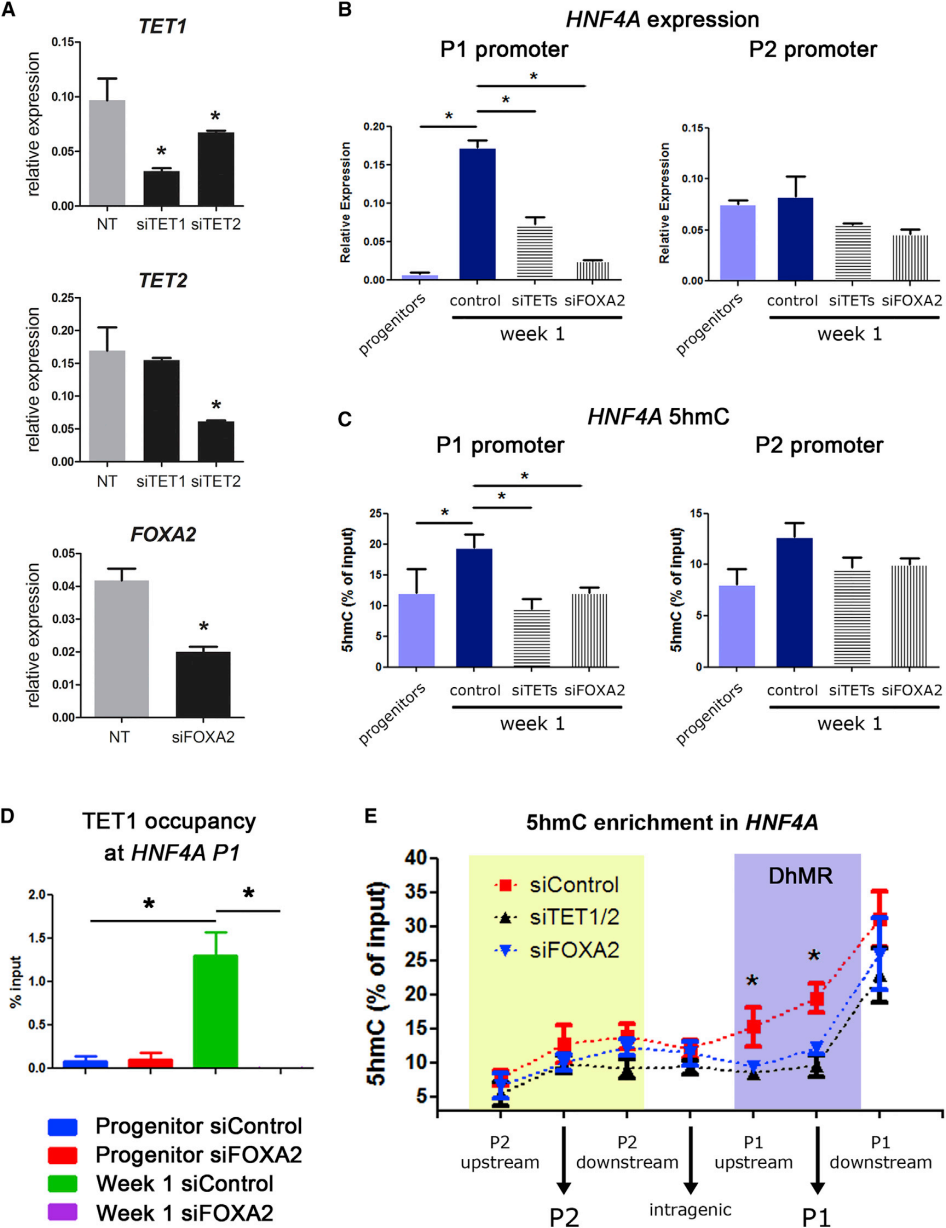
TET1 and FOXA2 Are Required for 5hmC and HNF4A Isoform Switch (A) siRNA silencing of *TETs* and *FOXA2* was done in HepaRG cells at the progenitor stage. Efficiency of silencing was assessed by qRT-PCR of *TETs* and *FOXA2* expression. (B) After 1 week of differentiation with each condition, RNA was extracted for assessment of P1 (left panel) and P2 (right panel) isoform expression by qRT-PCR. Proliferative HepaRG and control (non-targeting) siRNA are included in each bar plot. (C) Under the same siRNA conditions, DNA was extracted for quantification of 5hmC using hMedIP at the P1 (left panel) and P2 (right panel) promoters. (D) TET1 occupancy at P1 promoter was assessed by ChIP in control conditions (siControl) or after FOXA2 silencing (siFOXA2). (E) In an independent differentiation experiment, hMedIP was used to map 5hmC along the *HNF4A* gene, as shown in Figure 4A, under siRNA conditions. Results are shown after 1 week of differentiation for control (red), *TETs* silencing (black), and *FOXA2* silencing (blue). DhMR, differentially hydroxymethylated region. All assays were performed in at least three biological replicate experiments. Results are presented as means ± SD. ^∗^p < 0.05, t test.

After showing a differentiation-dependent colocalization between FOXA2 and TET1/2, we next hypothesized that these proteins are necessary for the promoter switch observed after 1 week of differentiation. We used small interfering RNAs (siRNAs) to transiently silence TETs or FOXA2 during the first days of progenitor differentiation (Figure 6A). As expected, the high 1-week induction of P1-driven *HNF4A* expression was significantly impaired after TET1/TET2 silencing (Figure 6B). An even more drastic shutdown of P1-driven expression was observed after FOXA2 silencing, while no changes in expression were observed in P2-driven isoforms (Figure 6B). As shown above, the 1-week induction of P1 isoforms was paralleled by a peak in 5hmC at the P1 DhMR (Figure 6C, left panel). In a similar way, the formation of such DhMR was impaired after silencing of TETs and FOXA2, with no significant changes in 5hmC at the P2 promoter (Figure 6C, right panel). Of note, no effect was observed after silencing of the related factor FOXA1 (Figure S5).

Based on the effect of FOXA2 silencing on 5hmC of HNF4A P1, and the known role of this protein as a pioneer TF during development, we hypothesized that FOXA2 was necessary for recruitment of TET1 to the P1 promoter. To test this notion, we performed ChIP assays during the first week of differentiation (when 5hmC takes place) with and without transient silencing of FOXA2 expression. First, we confirmed that TET1 occupancy is increased after 1 week of differentiation at the P1 locus (Figure 6D). In line with our hypothesis, this increase in occupancy is abolished in the presence of siRNAs against FOXA2 (Figure 6D). Finally, we mapped the changes in 5hmC along HNF4A in the presence and absence of TETs and FOXA2 transcripts (Figure 6E). We found that the week-1 increase in 5hmC is avoided by silencing TETs or FOXA2, and that this effect is limited to the region spanning the P1 promoter.

Therefore, TETs and FOXA2 are both required for the HNF4A expression switch that marks the early step of bipotent liver progenitor differentiation. These effects are limited to the P1 promoter region, in line with the P1 DhMR being an important regulatory region.

## Discussion

By profiling the methylome dynamics of liver progenitor differentiation, we have identified an epigenetic mark (5hmC) that signals the switch in isoform expression of a master TF of hepatocyte identity, HNF4A. We show that TET proteins, involved in such a signal, colocalize with the pioneer factor FOXA2. Finally, TETs and FOXA2 are both required for the 5hmC and the isoform switch that takes place during the first days of differentiation. A redistribution of methylation marks was also found in two additional in vitro models of hepatocyte differentiation (i.e., ESCs and iPSCs) and in human samples when comparing fetal and adult liver tissues.

In our working model, bipotent progenitors express P2-driven isoforms of HNF4A (dependent on the upstream promoter), while methylation of the downstream promoter impairs the expression of P1-driven isoforms. A 5hmC increase, leading to a P2-to-P1 switch in expression, is necessary for the commitment of a progenitor to the hepatocyte lineage. Once induced, P1 isoforms may be involved in the direct repression of the P2 promoter, as previously described (Briançon et al., 2004). FOXA2 may be necessary for TET1 recruitment to the P1 *HNF4A* locus in poised bipotent progenitors. Next, oxidation of methyl residues by TET1 proteins will lead to demethylation of the distal HNF4A promoter and the resulting expression of P1-driven isoforms. Therefore, modulation of 5hmC through interaction with TET proteins may represent a general mechanism of pioneer TFs. In line with this, and in addition to its well-known role in neural development (Santiago et al., 2014, Sun et al., 2014b), 5hmC and/or TETs have been recently implicated in key steps of terminal differentiation such as monocyte-to-macrophage differentiation (Wallner et al., 2016), specification of CD4 T cells (Nestor et al., 2016), cardiomyocyte development (Greco et al., 2016), and colonocyte differentiation (Chapman et al., 2015).

The association between a rather small change in methylation and an important transcriptional switch merits more detailed explanation. First, the bead array assay used to assess 5mC cannot distinguish between 5mC and 5hmC, and in some cases such as the P1 promoter, both DNA marks may display opposite patterns and therefore partially cancel out each other. Second, although we used differentiated populations with approximately 80% hepatocytes (based on total HNF4A protein expression), there is a mixed cell composition made of cholangiocytes and undifferentiated progenitor cells that introduce background levels of 5mC. The effect was clear enough to motivate the further analysis of 5hmC, which displayed a much more obvious effect (i.e., earlier appearance and higher in magnitude). In addition, results were confirmed with a technique (i.e., oxBS) that simultaneously measures 5mC and 5hmC in the same sample, and at base-resolution level (Figure S4). Finally, because of the intrinsic differences between template abundances (i.e., in general two DNA alleles for 5mC/5hmC versus hundreds of mRNA copies for qRT-PCR), significant methylation changes are usually translated in expression changes of higher magnitude.

Further studies will be required to dissect the interaction between FOXAs and TETs during hepatocyte differentiation. Of note, it was recently shown that another pioneer TF, FOXA1, physically interacts with the TET1 protein through its CXXC domain (Yang et al., 2016). In addition, studies should explore the mechanisms behind TET1 overexpression at the early stage of differentiation. Fitting with our observations, overexpression of TET1 was recently described as a response to cell confluence and/or inhibition of cell proliferation (Neri et al., 2015). In our hands, HepaRG progenitors reduce their mitotic index 48 times after only 1 week of differentiation, as assessed by counting the percentage of mitotic nuclei with DAPI staining (data not shown). Therefore, this may represent the initial trigger for TET1 overexpression.

We have shown that the epigenetic switch in promoter choice is a reversible process, an observation that may be relevant in pathological conditions where loss of hepatocyte identity is a well-known finding. For example, while differentiation of liver progenitors seems to be associated with a progressive disappearance of an inflammation-like state (Parent and Beretta, 2008), cytokines such as interleukin-6 and transforming growth factor β may be able to induce hepatocyte dedifferentiation (Cabillic and Corlu, 2016, Dubois-Pot-Schneider et al., 2014). Indeed, the importance of HNF4A in regulating inflammatory networks linked to liver and intestinal cancer has been recently highlighted (Babeu and Boudreau, 2014, Chahar et al., 2014, Tanaka et al., 2006). Interestingly, several studies also linked the different HNF4A isoforms to malignancy. Indeed, the expression of P1- and P2-driven HNF4A was described to be altered in several tumor tissues (Tanaka et al., 2006). In the context of hepatocellular carcinoma, HNF4A P1 isoforms were found to be downregulated (Tanaka et al., 2006). Other reports have shown that overexpression of P1-driven isoforms leads to morphological changes and reduced hepatocellular and renal cell carcinoma proliferation (Chiba et al., 2005, Lazarevich et al., 2004, Lucas et al., 2005). In this sense, understanding how the balance between P1 and P2 HNF4A isoforms is maintained will have implications in pathological contexts further to normal hepatocyte differentiation.

In summary, our data uncover the role of hydroxymethylation of *HNF4A* in the differentiation of a bipotent liver progenitor into hepatocytes. It supports a model whereby FOXA2 behaves as a pioneer factor required by TET proteins during this process (Wang et al., 2015).

## Experimental Procedures

### Immunofluorescence and In Situ Proximity Ligation Assay

Human HepaRG cells (Biopredic) were maintained and differentiated as previously described (Cerec et al., 2007, Gripon et al., 2002), and as depicted in Figure 1A. For immunofluorescence and PLA, HepaRG cells were plated on coverslips. At different time points cells were washed with PBS, fixed in 4% formaldehyde, and washed twice with PBS. Primary antibodies for immunofluorescence were anti-β-tubulin and anti-HNF4A (Table S2). After secondary antibodies, coverslips were washed and mounted on a slide with a mounting medium containing DAPI for nuclear counterstaining.

PLA was performed using the Duolink In Situ Kit (Sigma-Aldrich) following the manufacturer's recommendations. Fixed cells were incubated with specific primary antibody against FOXA2 protein and tested interactants (Table S2): HNF4, TET1, and TET2. Interactions were revealed using secondary antibodies coupled to specific PLA DNA probes that hybridized and were enzymatically joined when located in close proximity. After rolling circle amplification, each interaction generated a fluorescent spot that was analyzed under a fluorescence microscope (Nikon Eclipse Ti-E). Negative control was performed without primary antibodies. Cells were analyzed using a fluorescence microscope (Eclipse Ti, Nikon Instruments) and images were taken using NIS-Elements software (Nikon Instruments). ImageJ software was used for quantification of spots.

### qRT-PCR and siRNA Transfection

Total RNA was isolated using TRIzol reagent (Invitrogen), and reverse transcription reactions were performed using MMLV-RT (Invitrogen) and random hexamers, according to the manufacturer's protocol. Primers and probes were designed using Universal Probe Library Assay Design Center (Roche). qRT-PCR was performed in triplicates of each condition, using SyBR green (Eurogentec) and a CFX96 PCR system (Bio-Rad). *SFRS4* was used as housekeeping gene.

siRNA non-targeting and pool siRNAs against *FOXA2, TET1,* and *TET2* (Dharmacon, On-Target Plus siRNA) were transfected at the concentration of 20 nM using RNAiMAX lipofectamine (Life Technologies) as recommended by the manufacturer. Cells were washed and medium was replaced 12 hr after transfection.

### Bisulfite Modification, Pyrosequencing and Bead Array Methylation

To quantify the percentage of methylated cytosine in individual CpG sites, we performed bisulfite pyrosequencing as previously described (Hernandez-Vargas et al., 2010). For samples processed for Infinium bead arrays, the conversion was performed on 600 ng of DNA using the EZ DNA methylation Kit (Zymo Research) and modified DNA was eluted in 16 μL of water. Pyrosequencing assays (primers for PCR, sequencing primers and regions) are detailed in Table S3.

Methylation profiles were obtained with Humanmethylation450 Infinium bead arrays (Illumina), using recommended protocols for amplification, labeling, hybridization, and scanning. Each methylation analysis was performed in HepaRG cells differentiated in three independent wells at each time point.

### Bioinformatics Analysis

Raw methylation data were imported and processed using R/Bioconductor packages (Du et al., 2008, Pidsley et al., 2013). To define differentially methylated positions (DMPs) and differentially methylated regions (DMRs), we modeled the differentiation time points as a continuous variable in a linear regression using an empirical Bayesian approach (Smyth, 2004). DMPs were selected based on a differential methylation (delta beta) of at least 5% when comparing the first and last weeks of differentiation. DMRs were identified with the DMRcate package using the recommended proximity-based criteria (Peters et al., 2015) of at least 2 differentially methylated CpG sites with a maximum gap of 1,000 bp. All methylation data have been deposited in the GEO (accession number GEO: GSE72074).

For in vitro and in vivo in silico validations, data were downloaded from the GEO repository using accession numbers GEO: GSE66077 (Wilson et al., 2015) and GSE61278 (Bonder et al., 2014), respectively. Raw data (idat files) were imported to R and analyzed with R/Bioconductor packages, as described above. 5hmC detection blocks for adult and fetal livers were downloaded directly from the corresponding publication (Ivanov et al., 2013). Chromosomal annotations were used to evaluate the overlap between 5hmC blocks and the *HNF4A* locus using the GenomicRanges Bioconductor package, and to visualize the signal using UCSC.

### Immunoblotting, Chromatin Immunoprecipitation, and Hydroxymethyl Immunoprecipitation

Equal amounts of protein lysates (30–50 μg) were separated by SDS-PAGE and electrotransferred to Immobilon-P membranes (Millipore). Primary antibodies specific to P1- and P2-driven isoforms of HNF4A (R&D Systems) have been previously described (Chellappa et al., 2016) (Table S2).

For ChIP, cells were crosslinked with formaldehyde and chromatin was sheared using a Bioruptor sonicator (Diagenode). Assays were performed in triplicates of each condition with an SX-8G IP-Star automated system (Diagenode), using antibodies specific for FOXA2, TET1, TET2, and POL2A (Table S2). For hMedIP, we used antibody specific for 5hmC as well as spiked-in DNA standards against 5hmC, 5mC, and cytosine. For all ChIP and hMedIP experiments we applied isotype-specific immunoglobulin G (IgG) raised in the species as the primary antibodies. Primers used for ChIP and hMedIP are shown in Table S3. Results were calculated as percentage of the input for each condition, including the background IgG control antibody.

### Statistical Analysis

R/Bioconductor packages were used for bead array analyses, as described above. For other comparisons, means and differences of the means with 95% confidence intervals were obtained using GraphPad Prism (GraphPad Software). Mann-Whitney tests were used for unpaired analyses comparing average expression between classes. p Values of less than 0.05 were considered statistically significant. On each plot, SD represents the variation between three biological replicates.

## Author Contributions

P.-B.A., S.E., D.G., F.R.T., and D.M.N. performed the progenitor differentiation experiments. P.-B.A. performed sample preparations for bead array assays, and all validations. P.-B.A., M.-P.L., and M.-P.C. performed the PLA. S.E. and P.-B.A. performed the ChIP and siRNA experiments. F.R.T., V.C., and D.M.N. performed additional differentiation and validation experiments. A.C. and Z.H. provided conceptual assistance. P.-B.A. and H.H.-V. performed the statistical and bioinformatics analyses. A.C. and H.H.-V. supervised the experiments. H.H.-V. coordinated the project and wrote the manuscript. All authors discussed the results and manuscript text.

## Supplemental Information

Document S1. Supplemental Experimental Procedures, Figures S1–S5, and Tables S1–S3

Document S2. Article plus Supplemental Information
