# Supplementary material for: TET-Catalyzed 5-Hydroxymethylation Precedes HNF4A Promoter Choice during Differentiation of Bipotent Liver Progenitors
Source: Stem Cell Reports. 2017 Jun 22;9(1):264–78. doi: 10.1016/j.stemcr.2017.05.023 (PMC5511103; doi:10.1016/j.stemcr.2017.05.023)
Supplement: Document S1. Supplemental Experimental Procedures, Figures S1–S5, and Tables S1–S3 [file mmc1.pdf]

Stem Cell Reports, Volume *9*

Supplemental Information

TET-Catalyzed 5-Hydroxymethylation Precedes *HNF4A* Promoter Choice during Differentiation of Bipotent Liver Progenitors

Pierre-Benoit Ancey, Szilvia Ecsedi, Marie-Pierre Lambert, Fazlur Rahman Talukdar, Marie-Pierre Cros, Denise Glaise, Diana Maria Narvaez, Veronique Chauvet, Zdenko Herceg, Anne Corlu, and Hector Hernandez-Vargas

Supplemental Information

TET-catalyzed 5-hydroxymethylation precedes *HNF4A* promoter choice during differentiation of bipotent liver progenitors

Authors

Pierre-Benoit Ancey [1,#], Szilvia Ecsedi [1,2,#], Marie-Pierre Lambert [3], Fazlur Rahman Talukdar [1], Marie-Pierre Cros [1], Denise Glaise [4], Diana Maria Narvaez [1,5], Veronique Chauvet [1], Zdenko Herceg [1], Anne Corlu [4], Hector Hernandez-Vargas [1]

1. Epigenetics Group. International Agency for Research on Cancer (IARC), 150 Cours Albert-Thomas, 69008 Lyon, France.
2. MTA-DE Public Health Research Group, University of Debrecen, Debrecen, Hungary.
3. Epissage alternatif et progression tumorale. Centre de Recherche en Cancérologie de Lyon (CRCL), 28 rue Laennec, 69008 Lyon, France.
4. Inserm, UMR 991, Liver Metabolism and Cancer, Hôpital Pontchaillou, 35033 Rennes cedex; Université de Rennes 1, 35043 Rennes, France.
5. Human Genetics Laboratory, Department of Biological Sciences, Universidad de Los Andes, Bogotá, Colombia.

# Equal contribution

Supplementary Methods

Cell culture

HepaRG (Biopredic, Saint Grégoire, France) is a human bipotent cell line that can be differentiated into hepatocyte-like (Cerec et al., 2007) and cholangiocyte-like (Dianat et al., 2014) cells. HepaRG

cells were maintained and differentiated in culture as previously described (Cerec et al., 2007; Gripon et al., 2002), and as depicted in Fig 1a. Cells (6 × 104/well) were seeded in culture six-well plates using William's Medium E (Invitrogen, Carlsbad, CA, USA) and incubated at 37°C, 5% CO2. The medium was renewed every 3 days. Once a maximal confluence was reached (85 to 100%), culture medium was supplemented with epidermal growth factor (EGF, 90 ng/mL) during 1 week then with EGF and 2% dimethyl sulfoxide (DMSO) for another week to stimulate HepaRG cells differentiation. Both EGF and DMSO were kept thereafter until final differentiation (week 4). HepaRG cells were periodically tested for mycoplasma contamination.

Immunofluorescence

HepaRG cells were plated on coverslips and differentiated as described above. At different time points, cells were washed with PBS, fixed in 4% formaldehyde, and washed twice with PBS. Primary antibodies for immunofluorescence were anti-beta-tubulin and anti-HNF4A (Table S2). After secondary antibodies, coverslips were washed and mounted on a slide with a mounting medium containing DAPI for nuclear counterstaining. Cells were analyzed using a fluorescence microscope (Eclipse Ti, Nikon Instruments, Melville, NY, USA) and images were taken using the NIS-Elements software (NIS, Nikon Instruments).

Quantitative PCR (qRT-PCR)

Total RNA was isolated using the TRIzol Reagent (Invitrogen) according to the manufacturer's instructions. Reverse transcription reactions were performed using MMLV-RT (Invitrogen) and random hexamers, according to the manufacturer's protocol. Primers and probes were designed using Universal Probe Library Assay Design Center (Roche, Basel, Switzerland). qRT-PCR was performed in triplicates of each condition, using SyBR green (Eurogentec) and a CFX96 PCR system (Biorad). *SFRS4* was used as housekeeping gene.

Bisulfite modification and pyrosequencing

After trypsinization, cells were pelleted and resuspended in lysis buffer (1% SDS, 0.1M NaCl, 0.1M EDTA, 0.05M Tris pH8) with Proteinase K (500ug/ml) and incubated for 2 hours at 55°C. DNA was saturated with NaCl (6M), precipitated with isopropanol, and cleaned with 70% ethanol. Extracted DNA was finally resuspended in water. Quantity and quality of the extracted DNA were assessed with a ND-8000 spectrophotometer (Nanodrop, Thermo scientific). To quantify the percentage of methylated cytosine in individual CpG sites, we performed bisulfite pyrosequencing, as previously described (Hernandez-Vargas et al., 2010). For samples processed for Infinium bead arrays, the conversion was

performed on 600 ng of DNA using the EZ DNA methylation Kit (Zymo Research) and modified DNA was eluted in 16 ul of water. Quality of modification was checked by PCR using modified and unmodified primers for *GAPDH* gene. Pyrosequencing assays (primers for PCR, sequencing primers and regions) are described in Table S3.

Bead array methylation assays

Methylation profiles of the different samples were analyzed using the 450K Infinium methylation bead arrays (Illumina, San Diego, USA). Briefly, the Infinium Humanmethylation450 beadchip interrogates more than 480,000 methylation sites (Bibikova et al., 2011). The analysis on the bead array was conducted following the recommended protocols for amplification, labelling, hybridization and scanning. Each methylation analysis was performed in HepaRG cells differentiated in 3 independent wells at each time point.

Bioinformatics Analysis

Raw methylation data was imported and processed using R/Bioconductor packages (Du et al., 2008; Pidsley et al., 2013). Data quality was inspected using boxplots for the distribution of methylated and unmethylated signals, and inter-sample relationship using multidimensional scaling plots and unsupervised clustering. Probes were filtered for low quality (detection P value > 0.05) and known cross-reactive probes (Y. Chen et al., 2013). The remaining dataset was background substracted, and normalized using intra-array beta-mixture quantile normalization (Teschendorff et al., 2013). Methylation beta values were logarithmically transformed to M values before parametric statistical analyses, as recommended (Du et al., 2010). To define differentially methylated positions (DMPs) and differentially methylated regions (DMRs), we modelled the differentiation time points as a continuous variable in a linear regression using an empirical Bayesian approach (Smyth, 2004). DMPs were selected based on a differential methylation (delta beta) of at least 5% when comparing the first and last weeks of differentiation. DMRs were identified with the DMRcate package using the recommended proximity-based criteria (Peters et al., 2015). A DMR was defined by the presence of at least 2 differentially methylated CpG sites with a maximum gap of 1000 bp. Differentially methylated genes were further analyzed to determine functional pathways and ontology enrichment using Enrichr (E. Y. Chen et al., 2013). All methylation data have been deposited to the Gene Expression Omnibus repository (GEO accession number GSE72074).

For in vitro and in vivo in silico validations, data was downloaded from GEO repository, using accession numbers GSE66077 (Wilson et al., 2015) and GSE61278 (Bonder et al., 2014), respectively. Raw data (idat files) were imported to R and analyzed with R/Bioconductor packages, as

described above. 5hmC detection blocks for adult and foetal livers were downloaded directly from the corresponding publication (Ivanov et al., 2013). Chromosomal annotations were used to evaluate the overlap between 5hmC blocks and the *HNF4A* locus using the GenomicRanges Bioconductor package, and to visualize the signal using UCSC.

Chromatin Immunoprecipitation (ChIP) and hydroxymethyl-immunoprecipitation (hMedIP)

Cells were cross-linked with formaldehyde, and chromatin was sheared using a Bioruptor sonicator (Diagenode). Chromatin immunoprecipitation (ChIP) assays were performed in triplicates of each condition with an SX-8G IP-Star automated system (Diagenode), using antibodies specific for FOXA2, TET1, TET2, and POL2A (Table S3).

Purified genomic DNA was sonicated using a Bioruptor (Diagenode) to obtain fragments of 300–700 bp. Denatured DNA samples were subjected to hMeDIP using an automated (IP-Star) system. Following the manufacturer's recommendations (Diagenode), we used antibody specific for 5hmC as well as spiked-in DNA standards against 5hmC, 5mC and cytosine. For all the ChIP and hMeDIP experiments we applied isotype specific IgG raised in the species as the primary antibodies.

Primers used for ChIP and hMedIP are shown in Table 4. Results were calculated as percentage of the input for each condition, including the background IgG control antibody.

Immunoblotting

Equal amounts of protein lysates (30–50 µg) were separated by sodium dodecyl sulfate – polyacrylamide gel electrophoresis and electrotransferred to Immobilon-P membranes (Millipore Corporation, Bedford, MA, USA). Primary antibodies specific to P1- and P2-driven isoforms of HNF4A (R&D Systems) have been previously described (Chellappa et al., 2016), and are listed in Table S2. After incubation with peroxidase-conjugated secondary antibody (Dako, Glostrup, Denmark), protein expression was detected using ECL Western blotting reagents (Amersham Biosciences, GE Healthcare, London, UK).

In situ Proximity Ligation Assay

HepaRG were differentiated for 1 week as previously described. The in situ proximity ligation assay (PLA) was performed using the Duolink in Situ Kit (Sigma Aldrich) following manufacturer's recommendations. Briefly, fixed cells (non-differentiated and differentiated HepaRG) were incubated with specific primary antibody against FOXA2 protein and tested interactants (Table S2): HNF4, TET1

and TET2. Interactions were revealed using secondary antibodies coupled to specific PLA DNA probes that hybridized and were enzymatically joined when located in close proximity. After rolling circle amplification, each interaction generated a fluorescent spot that was analyzed by fluorescence microscopy (Nikon Eclipse Ti-E). Negative control was performed without primary antibodies. ImageJ software was used for quantification of spots.

siRNA transfection

siRNA non-targeting and pool siRNAs against *FOXA2, TET1* and *TET2* (Dharmacon, On-Target plus siRNA) were transfected at the concentration of 20nM using RNAiMAX lipofectamine (LifeTechnologies) as recommended by the manufacturer. Cells were washed and medium was replaced 12 hours after transfection.

Oxidative bisulfite conversion (OxBS)

Oxidative bisulfite conversion reactions were done using the CEGX True Methyl kit (Cambridge Epigenetix). One 1 µg of DNA from all samples was purified and denatured. DNA from each subject was then split in two equal reactions, one of which underwent chemical oxidation followed by bisulfite conversion (oxBS), the other underwent mock oxidation (oxidant replaced by water) followed by bisulfite conversion (BS). Subsequent PCR and pyrosequencing reactions were performed as described for bisulfite pyrosequencing. Cytosines derived from oxBS treatment represent “true” 5mC levels. Therefore, subtraction of BS-oxBS shows 5hmC whereas the level of oxBS itself represents 5mC.

Statistical Analysis

R/Bioconductor packages were used for bead array analyses, as described above. For other comparisons, means and differences of the means with 95% confidence intervals were obtained using GraphPad Prism (GraphPad Software Inc.). Mann-Whitney tests were used for unpaired analyses comparing average expression between classes. P values < 0.05 were considered statistically significant. On each plot standard deviation represents the variation between three biological replicates.

References

Bibikova, M., Barnes, B., Tsan, C., Ho, V., Klotzle, B., Le, J.M., Delano, D., Zhang, L., Schroth, G.P., Gunderson, K.L., Fan, J.-B., Shen, R., 2011. High density DNA methylation array with single CpG site resolution. Genomics 98, 288–295. doi:10.1016/j.ygeno.2011.07.007

Bonder, M.J., Kasela, S., Kals, M., Tamm, R., Lokk, K., Barragan, I., Buurman, W.A., Deelen, P., Greve, J.-W., Ivanov, M., Rensen, S.S., van Vliet-Ostaptchouk, J.V., Wolfs, M.G., Fu, J., Hofker, M.H., Wijmenga, C., Zhernakova, A., Ingelman-Sundberg, M., Franke, L., Milani, L., 2014. Genetic and epigenetic regulation of gene expression in fetal and adult human livers. BMC Genomic- 15. doi:10.1186/1471-2164-15-860

Cerec, V., Glaise, D., Garnier, D., Morosan, S., Turlin, B., Drenou, B., Gripon, P., Kremsdorf, D., Guguen-Guillouzo, C., Corlu, A., 2007. Transdifferentiation of hepatocyte-like cells from the human hepatoma HepaRG cell line through bipotent progenitor. Hepatol. 45, 957–967. doi:10.1002/hep.21536

Chellappa, K., Deol, P., Evans, J.R., Vuong, L.M., Chen, G., Briançon, N., Bolotin, E., Lytle, C., Nair, M.G., Sladek, F.M., 2016. Opposing roles of nuclear receptor HNF4α isoforms in colitis and colitis-associated colon cancer. eLife 5. doi:10.7554/eLife.10903

Chen, E.Y., Tan, C.M., Kou, Y., Duan, Q., Wang, Z., Meirelles, G.V., Clark, N.R., Ma’ayan, A., 2013. Enrichr: interactive and collaborative HTML5 gene list enrichment analysis tool. BMC Bioinforma. 14. doi:10.1186/1471-2105-14-128

Chen, Y., Lemire, M., Choufani, S., Butcher, D.T., Grafodatskaya, D., Zanke, B.W., Gallinger, S., Hudson, T.J., Weksberg, R., 2013. Discovery of cross-reactive probes and polymorphic CpGs in the Illumina Infinium HumanMethylation450 microarray. Epigenetics: Off. J. DNA Methylation Soc. doi:10.4161/epi.23470

Dianat, N., Dubois-Pot-Schneider, H., Steichen, C., Desterke, C., Leclerc, P., Raveux, A., Combettes, L., Weber, A., Corlu, A., Dubart-Kupperschmitt, A., 2014. Generation of functional cholangiocyte-like cells from human pluripotent stem cells and HepaRG cells. Hepatol. 60, 700–714. doi:10.1002/hep.27165

Du, P., Kibbe, W.A., Lin, S.M., 2008. lumi: a pipeline for processing Illumina microarray. Bioinforma. 24, 1547–1548. doi:10.1093/bioinformatics/btn224

Du, P., Zhang, X., Huang, C.-C., Jafari, N., Kibbe, W.A., Hou, L., Lin, S.M., 2010. Comparison of Beta-value and M-value methods for quantifying methylation levels by microarray analysis. BMC Bioinforma. 11. doi:10.1186/1471-2105-11-587

Gripon, P., Rumin, S., Urban, S., Le Seyec, J., Glaise, D., Cannie, I., Guyomard, C., Lucas, J., Trepo, C., Guguen-Guillouzo, C., 2002. Infection of a human hepatoma cell line by hepatitis B virus. Proc. Natl. Acad. Sci. United States Am. 99, 15655–15660. doi:10.1073/pnas.232137699

Hernandez-Vargas, H., Lambert, M.-P., Le Calvez-Kelm, F., Gouysse, G., McKay-Chopin, S., Tavtigian, S.V., Scoazec, J.-Y., Herceg, Z., 2010. Hepatocellular carcinoma displays distinct DNA methylation signatures with potential as clinical predictors. PLoS ONE 5. doi:10.1371/journal.pone.0009749

Ivanov, M., Kals, M., Kacevska, M., Barragan, I., Kasuga, K., Rane, A., Metspalu, A., Milani, L., Ingelman-Sundberg, M., 2013. Ontogeny, distribution and potential roles of 5-hydroxymethylcytosine in human liver function. Genome Biol. 14. doi:10.1186/gb-2013-14-8-r83

Peters, T.J., Buckley, M.J., Statham, A.L., Pidsley, R., Samaras, K., V Lord, R., Clark, S.J., Molloy, P.L., 2015. De novo identification of differentially methylated regions in the human genome. Epigenetics & chromatin 8. doi:10.1186/1756-8935-8-6

Pidsley, R., Y Wong, C.C., Volta, M., Lunnon, K., Mill, J., Schalkwyk, L.C., 2013. A data-driven approach to preprocessing Illumina 450K methylation array data. BMC Genomic- 14. doi:10.1186/1471-2164-14-293

Smyth, G.K., 2004. Linear models and empirical bayes methods for assessing differential expression in microarray experiments. Stat. Appl. Genet. Mol. Biol. doi:10.2202/1544-6115.1027

Teschendorff, A.E., Marabita, F., Lechner, M., Bartlett, T., Tegner, J., Gomez-Cabrero, D., Beck, S., 2013. A beta-mixture quantile normalization method for correcting probe design bias in Illumina Infinium 450 k DNA methylation data. Bioinforma. doi:10.1093/bioinformatics/bts680

Wilson, A.A., Ying, L., Liesa, M., Segeritz, C.-P., Mills, J.A., Shen, S.S., Jean, J., Lonza, G.C., Liberti, D.C.,

Lang, A.H., Nazaire, J., Gower, A.C., Müeller, F.-J., Mehta, P., Ordóñez, A., Lomas, D.A., Vallier, L., Murphy, G.J., Mostoslavsky, G., Spira, A., Shirihai, O.S., Ramirez, M.I., Gadue, P., Kotton, D.N., 2015. Emergence of a stage-dependent human liver disease signature with directed differentiation of alpha-1 antitrypsin-deficient iPS cells. Stem cell reports 4, 873–885. doi:10.1016/j.stemcr.2015.02.021

Supplementary Tables

Table S1. Differentially methylated regions (DMRs).
DNA methylation was performed at the single-site level (differentially methylated positions or DMPs) and at the region level (DMRs). DMPs are listed in Table 1. Although there is a partial overlap between DMPs and DMRs, they use different statistics (see Experimental Procedures).

Symbol	Group	Genomic location	No. of probes	mean pval	Size (bp)
HNF4A	TSS1500	chr20:43029468-43029997	8	5.186E-07	529
EHMT2	Body	chr6:31859141-31859187	2	2.024E-06	46
DHCR24	Body	chr1:55331285-55331361	2	1.978E-05	76
PXMP4	Body	chr20:32307885-32308529	11	3.358E-05	644
F2	TSS1500	chr11:46740481-46740790	4	5.385E-05	309
PTPN22	Body	chr1:114414042-114414802	5	0.0001785	760
DEFB125	TSS200	chr20:68170-68396	2	0.0001806	226
C22orf9,MIR1249	Body	chr22:45596797-45597767	10	0.0001947	970
KIAA1217	5'UTR	chr10:24496598-24496943	3	0.0002192	345
RIN2	Body	chr20:19955436-19955868	4	0.0003367	432
C10orf26	TSS1500	chr10:104535276-104536695	12	0.0004738	1419
ARVCF	Body	chr22:19960832-19961060	3	0.0005719	228
GPT	TSS1500	chr8:145728138-145728630	13	0.0006806	492
FAM49A	5'UTR	chr2:16804409-16805111	4	0.0009913	702
		chr16:1349406-1350103	4	0.0011129	697
ZNF407	1stExon	chr18:72342942-72342999	2	0.0015105	57
SERPINA1	1stExon	chr14:94856984-94857275	4	0.0020782	291
DIRC3	Body	chr2:218465569-218466469	4	0.0022271	900
KIAA1199	TSS1500	chr15:81070851-81071708	3	0.0022973	857
		chr1:26490989-26491047	2	0.0034244	58
LOC645323	Body	chr5:87973439-87973597	2	0.0036908	158
SCAMP5	TSS1500	chr15:75287447-75288289	8	0.0037323	842
BAT2	Body	chr6:31596847-31597461	8	0.004334	614
NEIL2	TSS1500	chr8:11625764-11625883	2	0.0061087	119
RND2	TSS1500	chr17:41176469-41176864	4	0.0062863	395
PPM1H	Body	chr12:63193647-63194001	2	0.0066949	354
CD96	TSS1500	chr3:111259817-111259879	2	0.008184	62
		chr18:71982691-71982907	2	0.0086344	216
ZNF804A	1stExon	chr2:185463132-185463803	6	0.0096172	671
NFIC	Body	chr19:3369478-3370244	6	0.0097666	766
GAK	Body	chr4:887730-888127	2	0.0098505	397
		chr19:59050122-59050280	2	0.0108477	158
GABRR2	TSS200	chr6:90025032-90025307	4	0.0114087	275
GHRLOS,C3orf42	TSS1500	chr3:10326540-10326774	3	0.0115875	234
KRT1	TSS1500	chr12:53075359-53075482	3	0.0119929	123
ZFPM2	TSS1500	chr8:106330170-106332188	12	0.0122865	2018
HDAC4	Body	chr2:240090592-240091133	5	0.0130066	541
CHI3L1	TSS200	chr1:203155938-203156625	5	0.0132376	687
ZNF551	TSS1500	chr19:58193071-58193426	10	0.013365	355
MYO5A	TSS1500	chr15:52822439-52822582	2	0.0134198	143
		chr8:142287226-142287264	2	0.0138458	38

Symbol	Group	Genomic location	No. of probes	mean pval	Size (bp)
C11orf9,DKFZP434K028	Body	chr11:61522002-61522498	3	0.0145227	496
RBM24	1stExon	chr6:17282354-17283113	9	0.014549	759
MIOS	5'UTR	chr7:7607013-7607056	3	0.0183682	43
MIR591,SLC25A13	TSS200	chr7:95849192-95849578	3	0.0194258	386
		chr1:47999516-47999856	2	0.0205292	340
KCNK1	TSS1500	chr1:233749381-233750313	10	0.0213053	932
DOCK6	TSS1500	chr19:11374363-11374482	2	0.0216775	119
TPM3	TSS1500	chr1:154164951-154164994	2	0.0220847	43
FAM103A1	TSS1500	chr15:83654517-83654607	3	0.0229223	90
PTPRT	TSS200	chr20:41818574-41819125	6	0.0230105	551
TREH	1stExon	chr11:118550379-118550644	5	0.0240656	265
AQP9	TSS200	chr15:58430391-58430539	2	0.0255012	148
CREM	Body	chr10:35484533-35484972	6	0.0257341	439
ZNF433	Body	chr19:12145947-12146509	5	0.026556	562
CTDP1	Body	chr18:77499754-77499769	2	0.0274301	15
CRISP2	TSS1500	chr6:49681742-49681774	2	0.0282112	32
ZNF213	5'UTR	chr16:3185452-3185592	2	0.0287104	140
MRGPRE	5'UTR	chr11:3250765-3250827	2	0.0290696	62
PRDM8	TSS1500	chr4:81105355-81105375	3	0.0304332	20
IQSEC1	3'UTR	chr3:12940286-12940432	3	0.0304607	146
LOC149134	TSS200	chr1:246952889-246952914	2	0.0318531	25
SH3GL3	TSS200	chr15:84115895-84116151	8	0.0320566	256
SHANK2	Body	chr11:70416238-70416309	3	0.0334534	71
RING1	Body	chr6:33177497-33177949	13	0.0345594	452
ITIH3	TSS1500	chr3:52827657-52827704	2	0.0365981	47
GEFT	TSS1500	chr12:58003898-58004248	6	0.0377622	350
OR52W1	TSS1500	chr11:6219573-6219621	2	0.0431963	48
RPS6KA2	Body	chr6:166851830-166851891	2	0.0446485	61
NRXN2	Body	chr11:64458846-64458869	2	0.0471847	23
OR1G1	TSS1500	chr17:3031354-3031450	2	0.0490965	96

DMRs (minimum P value < 0.05) are classified according to their gene-centric distribution as 200 or 1500 bp from the transcription start site (TSS200 and TSS1500, respectively), gene body, or UTR (3' or 5'). Genomic location is based on hg19 coordinates.

Table S2. List of antibodies used for immunofluorescence (IF), proximity ligation assays (PLA), western blot (WB), and chromatin immunoprecipitation (ChIP).

Antibody	Origin	Company	catalog #	dilution for PLA/IF/WB	Quantity for ChIP (µg)	Application
Alexa 488	goat	Life technologies	A31627/A31619	1/100	NA	IF, FACS
Alexa 555	goat	Life technologies	A31621/A31629	1/100	NA	IF, FACS
b-tubulin	mouse	Sigma	T4026	1/100	NA	IF
FOXA2	mouse	Abcam	ab60721	1/100	2.5	PLA, ChIP
FOXA2	rabbit	Abcam	ab108422	1/300	2.5	PLA, ChIP
HNF4	rabbit	Cell signalling	3113S	1/100	NA	IF,PLA
HNF4-P1	mouse	R&D Systems	PP-K9218-00	1/500	NA	WB
HNF4-P2	mouse	R&D Systems	PP-H6939-00	1/500	NA	WB
POL2A	mouse	Thermo scientific	MA1-46093	NA	2.5	ChIP
TET1	rabbit	Active modif	61443	1/100	2.5	PLA, ChIP
TET2	rabbit	Genetex	GTX124205	NA	2.5	ChIP
5-hmC	rat	Diagenode	C15220001	NA	1.6	hMedIP

Table S3. List of HNF4A primers used for chromatin immunoprecipitation (ChIP), hMedIP and pyrosequencing.

Region	Sequence	Location (hg19)
**P1**	Fw (5′-ATCTTCCCAGAGGACGGTTT-3′)<br>Rv (5′-TGTAGGCTGGGTCCAGTG-3′)	chr20:43029881-43030082
**P1 upstream**	Fw (5’-GCCTGCCTTGTACAATTGATAACT-3’)<br>Rv (5’-TTGGCCTAGCCTCTGTGAA-3’)	chr20:43029665-43029868
**P1 downstream**	Fw (5’-CGATGGGCAATGGTAGGT-3’)<br>Rv (5’-CACCCAGAATGCCTGTGAT-3’)	chr20:43030116-43030320
**Intragenic**	Fw (5’-CTGAAGCAGGTCAAGAATCCA-3’)<br>Rv (5’- AAGTCAGGAGGAGGATGAG-3’)	chr20:43019651-43019855
**P2**	Fw (5′-ACTTCCTGCATGGTGACACA-3′)<br>Rv (5′-GTCCTGCACTCTGGGGTTAG-3′)	chr20:42984014-42984175
**P2 upstream**	Fw (5’-TGGTACATAGTAGGTGCTCAATAA-3’)<br>Rv (5’-AAAGGAGGGTGGAGAACTG-3’)	chr20:42983781-42983982
**P2 downstream**	Fw (5’-TTGGACTCTCACCTCTCCA-3’)<br>Rv (5’-GTAACCAGTCACTTAGGGAACC-3’)	chr20:42984067-42984268
**P1 pyrosequencing**	Fw: (5’-GGTGAGTTAGGGTTTTAGTAGTTG-3’)<br>Rv: (5’-AATCRCATTCTCCCTACCTCCAC-3’)<br>Sequencing: (5’-GATTTTTAGTAGATTTTTTTAGAGGA-3’)	
**P2 pyrosequencing**	Fw: (5’-TGGTTGTGTTGTTGTTGTGAG-3’)<br>Rv: (5’-CCCATAACCTCCCAAAACTAAC-3’)<br>Sequencing: (5’-AGATTGGATAGG-3’)	

Supplementary Figures

Figure S1. Technical validation of 5mC changes during HepaRG differentiation.

(**A**) Quantitative pyrosequencing was used to validate the methylation changes of the *HNF4A* CpG site indicated in Fig 1E (cg03862380). Each bar represents one time point of differentiation. (**B**) Technical validation by pyrosequencing of four additional loci found differentially methylated (Table 1). For each CpG site, 5mC content was assessed for progenitors (week 0) and its derived differentiated hepatocytes (week 4). (*) indicates t-test p value below 0.05. See also Figure 1.

Figure S2. Functional validation of 5mC changes during differentiation to hepatocytes.

(**A**) Heatmap and unsupervised clustering of ESCs and iPSCs samples based on all CpG sites mapping to *HNF4A*. Three time points of differentiation are included, as previously described (Wilson et al., 2015). Samples cluster by time point, regardless of the in vitro model system (i.e. ESCs or iPSCs). (**B**) Infinium 450k data during differentiation of induced pluripotent stem cells (iPSCs) towards hepatocytes. Cells were obtained at different time points (days 0, 5, and 24), as previously described (Wilson et al., 2015). 5mC values for all HNF4A CpG sites, as shown in (A) and Fig 2C. P1 promoter is shown in blue, and P2 promoter in yellow. DMR = differentially methylated region. See also Figure 2.

Figure S3. HNF4A isoform switch during hepatocyte differentiation.

Densitometry and quantification of HNF4A protein expression, using P1 and P2 specific antibodies, corresponding to HepaRG differentiation (**A**) and dedifferentiation (**B**). Corresponds to Figs 3D and 3F, respectively. Protein signal was and quantified using Image Studio Lite software. Bars show mean and standard deviation from three independent biological replicates. See also Figure 3.

Figure S4. 5mC in foetal vs. adult human liver.

(**A**) qRT-PCR expression of *TET1* (left panel) and *TET2* (right panel) at different time points of HepaRG differentiation. Only *TET1* was significantly different when comparing weeks 0 and 1 of differentiation. All values are relative to the housekeeping gene (*HPRT1*). (**B**) Oxidative bisulfite assay (oxBS) was used for independent validation of 5hmC and 5mC changes between progenitors (week zero) and differentiating (week one) HepaRG cells. 5hmC/5mC is shown for HNF4A P1 (upper panel) and P2 (bottom panel) promoters. (*) indicates t-test p value below 0.05. (**C**) 5mC data for foetal and adult liver tissues (Bonder et al., 2014) was reanalyzed, as described in Experimental Procedures. Unsupervised clustering of foetal (n=14) and adult (n=96) samples, based on 5mC at the *HNF4A*

locus. (**D**) 5mC data was used to visualize methylation (beta values) comparing the two tissue types, for the same locus shown in Fig 2. P1 promoter is shown in blue, and P2 promoter in yellow. DMR = differentially methylated region. See also Figure 4.

Figure S5. FOXA2 is required for and HNF4A expression switch.

siRNA silencing was performed to compare FOXA1 vs. FOXA2 role in HNF4A P1 switch. Efficiency of FOXA2 silencing was assessed by western blot (**A**), including also siRNA against TETs as an additional control. siRNA against both FOXA1 and FOXA2 was also assessed by qRT-PCR expression (**B**). (**C**) After one week of differentiation with each condition, RNA was extracted for assessment of P1 (left panel) and P2 (right panel) isoform expression by qRT-PCR. Proliferative HepaRG and control (non-targeting) siRNA are included in each bar plot. (*) indicates t-test p value below 0.05. See also Figure 6.

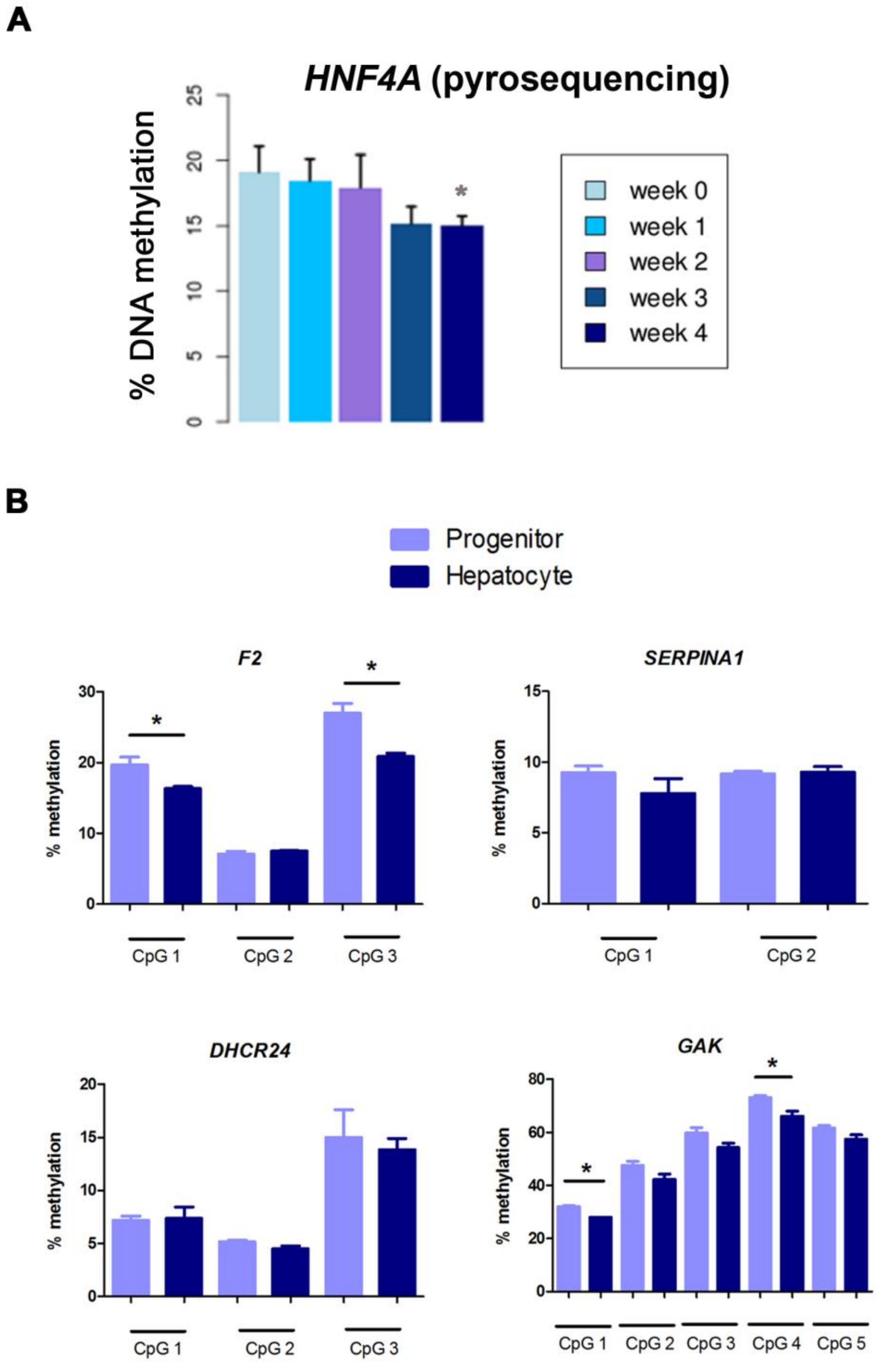

Figure S1. Technical validation of 5mC changes during differentiation to hepatocytes.

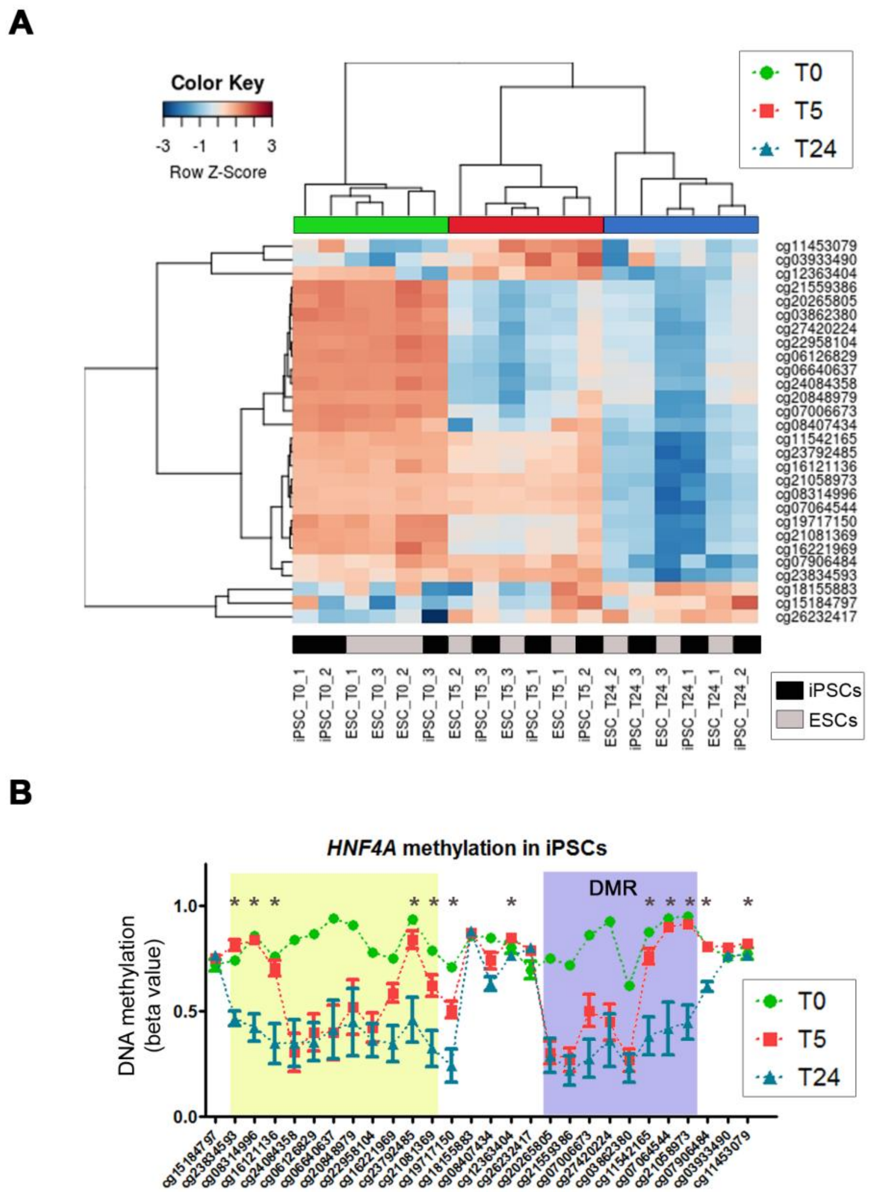

Figure S2. Functional validation of 5mC changes during differentiation to hepatocytes.

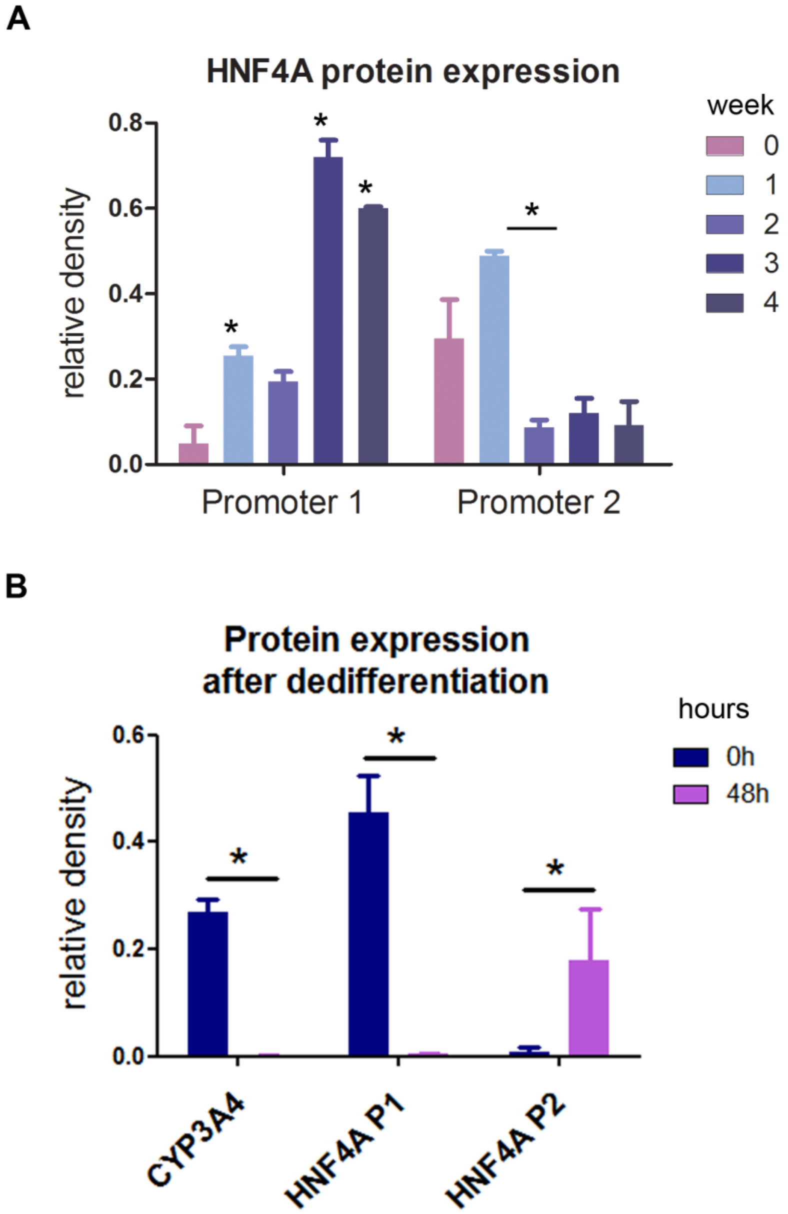

Figure S3. HNF4A isoform switch during hepatocyte differentiation.

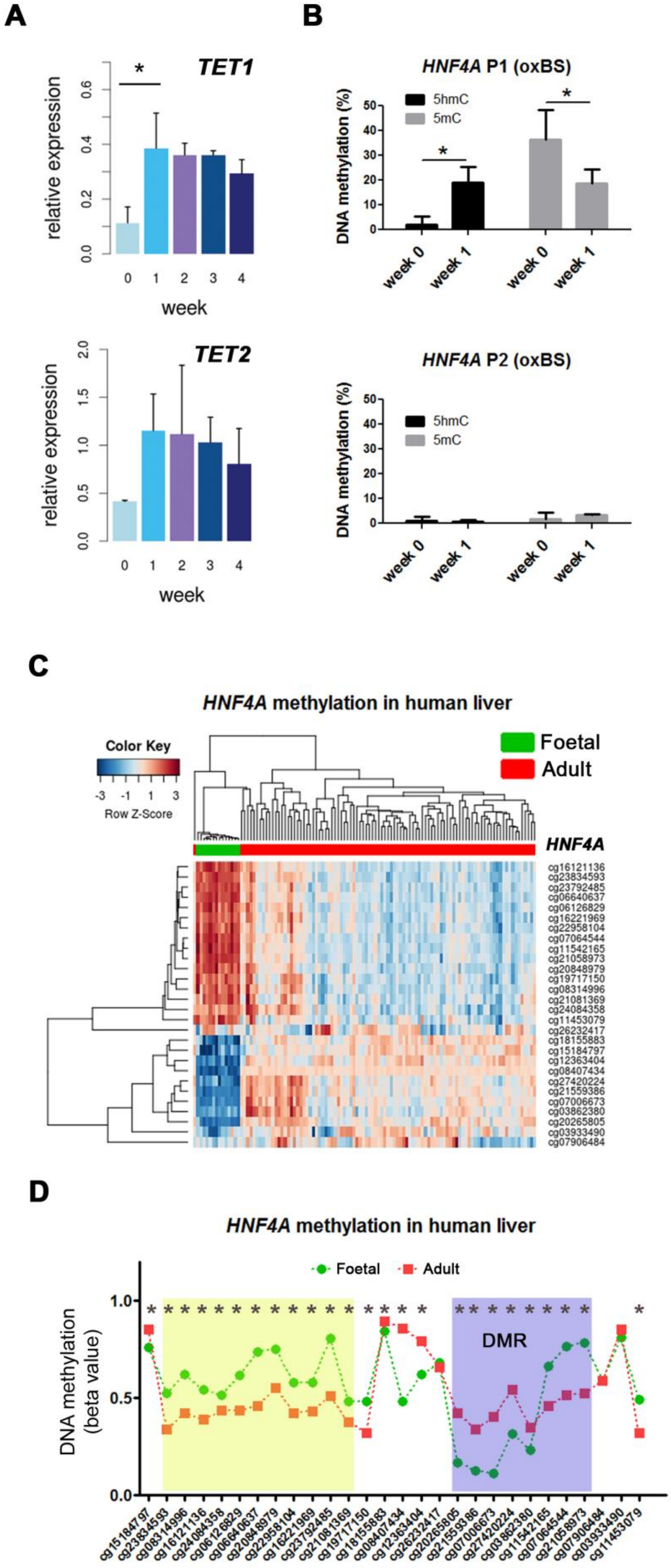

Figure S4. Hepatocyte methylation (5mC/5hmC) in vivo and in vitro.

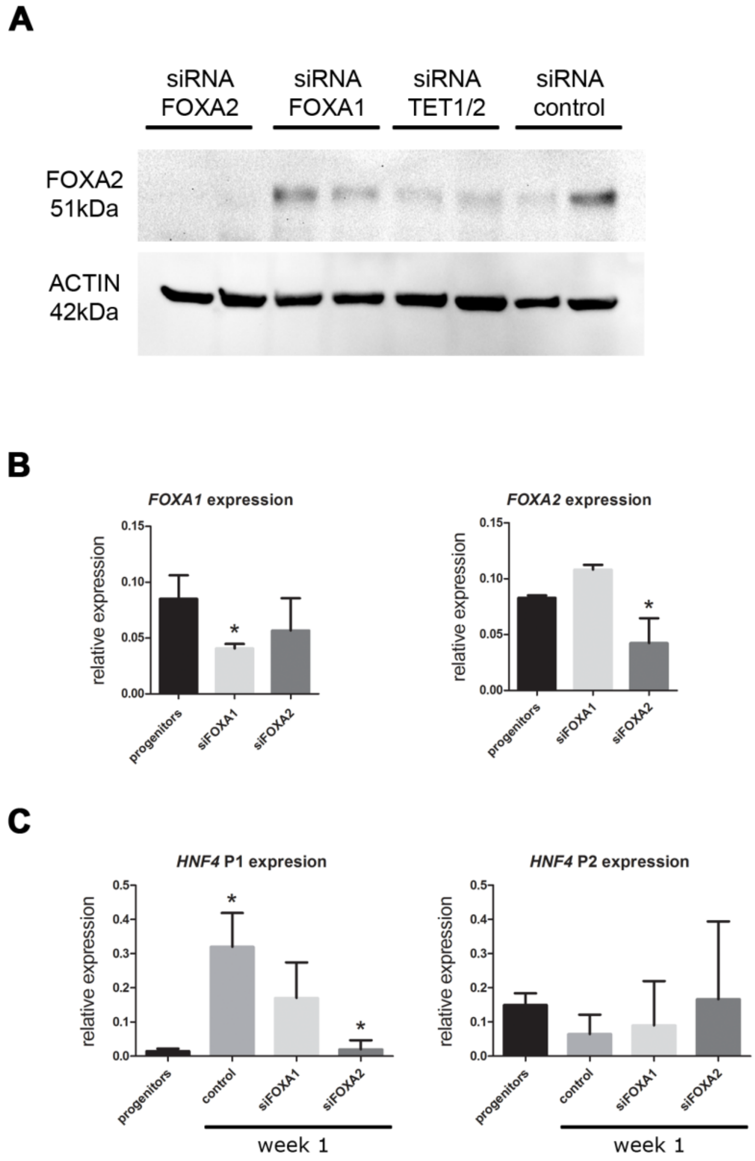

Figure S5. FOXA2 is required for HNF4A P1 expression switch.
